# An automatic and personalized recommendation modelling in activity eCoaching with deep learning and ontology

**DOI:** 10.1038/s41598-023-37233-7

**Published:** 2023-06-22

**Authors:** Ayan Chatterjee, Andreas Prinz, Michael Alexander Riegler, Yogesh Kumar Meena

**Affiliations:** 1 Department of Information and Communication Technology, University of Agder, 4879 Grimstad, Norway; 2 Department of Holistic Systems, Simula Metropolitan Center for Digital Engineering, Pilestredet 52, 0167 Oslo, Norway; 3 Department of Computer Science and Engineering & Centre for Cognitive and Brain Science, IIT Gandhinagar, Gandhinagar, India

**Keywords:** Health services, Quality of life

## Abstract

Electronic coaching (eCoach) facilitates goal-focused development for individuals to optimize certain human behavior. However, the automatic generation of personalized recommendations in eCoaching remains a challenging task. This research paper introduces a novel approach that combines deep learning and semantic ontologies to generate hybrid and personalized recommendations by considering “Physical Activity” as a case study. To achieve this, we employ three methods: time-series forecasting, time-series physical activity level classification, and statistical metrics for data processing. Additionally, we utilize a naïve-based probabilistic interval prediction technique with the residual standard deviation used to make point predictions meaningful in the recommendation presentation. The processed results are integrated into activity datasets using an ontology called OntoeCoach, which facilitates semantic representation and reasoning. To generate personalized recommendations in an understandable format, we implement the SPARQL Protocol and RDF Query Language (SPARQL). We evaluate the performance of standard time-series forecasting algorithms [such as 1D Convolutional Neural Network Model (CNN1D), autoregression, Long Short-Term Memory (LSTM), and Gated Recurrent Units (GRU)] and classifiers [including Multilayer Perceptron (MLP), Rocket, MiniRocket, and MiniRocketVoting] using state-of-the-art metrics. We conduct evaluations on both public datasets (e.g., PMData) and private datasets (e.g., MOX2-5 activity). Our CNN1D model achieves the highest prediction accuracy of 97$\%$, while the MLP model outperforms other classifiers with an accuracy of 74$\%$. Furthermore, we evaluate the performance of our proposed OntoeCoach ontology model by assessing reasoning and query execution time metrics. The results demonstrate that our approach effectively plans and generates recommendations on both datasets. The rule set of OntoeCoach can also be generalized to enhance interpretability.

## Introduction

The collaborative effects of sedentary lifestyle patterns are linked to multiple adverse health outcomes, including increased risk of lifestyle diseases such as obesity, type 2 diabetes, hypertension, depression, and cardiovascular disease^1–4^. Regular physical exercise positively affects the prevention and management of lifestyle diseases. People who are not physically active have a 20–30% increased risk of death compared to those who are physically active^5–8^. E-health research can improve personal healthcare through information and communication technology (ICT)^9,10^. eHealth technologies help collaborate and share health information through digital sensors for ubiquitous monitoring and care. eCoach systems can enable people to lead a healthy lifestyle through ubiquitous personalized health status monitoring (e.g., physical activity, diet, healthy habits) and personalized recommendation generation^11–13^.

An eCoach system is complex system with many partially connected computerized components interacting through various feedback loops. It creates an artificial entity that can sense, judge, learn and predict the behavior of individuals. It proactively engages in ongoing collaborative dialogue with individuals to support planning and encourage effective goal management through persuasive skills^11^. The eCoach system can generate automatic and customized activity recommendations based on insights from activity sensor data such as that collected using wearable Bluetooth activity devices such as Fitbit, MOX2-5, Garmin, and Actigraph for daily, weekly, or monthly activity goals. The activity coaching process can be face-to-face or technology-driven^11^. Personal coaching with manual activity tracking and generating recommendations is time-consuming and repetitious.

Recommendation technology can be defined as a decision-making approach in complex information environments^14–16^. The techniques can be classified as rule-based and data-driven^17^. Solely data-driven recommendation technology with machine learning (ML) and deep learning (DL) algorithms suffers from insufficient data, high computing overhead, lack of interpretability, re-training, personalization, and cold-start problem^17,18^. In contrast, a rule-based recommendation technology uses binary logic in a symbolic form to present knowledge in “IF-THEN or IF-ELSE IF-THEN” rules and infer new knowledge with reasoning. A knowledge base (KB) is retained to store and access such rules and related messages. Rules can be specified differently, such as propositional logic, decision tree, relational algebra, and description logic. Rule-based systems are modular, intelligible, and easy to manage; however, they suffer from symbol grounding problems^17^. Therefore, a hybrid approach may overcome the shortcomings of both data-driven and rule-based recommendation technologies.

Description logics (i.e., formal knowledge representation of ontology language) balance transparency, complexity, and effectiveness of knowledge description and knowledge reasoning. Moreover, semantic web rule language (SWRL) and SPARQL languages also represent description logics in an ontology^3,19,20^. In particular, ontology is a formal description of knowledge in a domain and its relationships according to a hierarchical structure, which can help existing technologies develop new ideas through conceptual modeling or proof-of-concept (PoC) research to address the challenges of semantic processing modeling. Unlike taxonomies or relational database schema, ontologies express relationships and allow users to connect or relate multiple concepts innovatively using the following elements: individuals/objects, classes, attributes, relations, and axioms^3,21^. They follow an open-world hypothetical knowledge representation style using the Web Ontology Language (OWL), Resource Description Framework (RDF), and RDF Schema (RDFS) syntax^3^. In addition, knowledge representation can be optimized by the ontology model, and the ontology reasoning engine can verify the stability of its logic and structure.

A digital activity recommendation system includes a data collection module, data processing and a recommendation generation or decision-making module. Data can be collected over time and analyzed using ML, DL, or rule-based algorithms to generate real-time feedback to achieve individual activity goals. The decision engine recommends changes to a person’s behavior, daily routine, and activity schedule. The eCoach feature can show hope and motivation to improve physical activity using wearable activity sensors and digital activity trackers. Various mobile applications for activity monitoring and lifestyle guidance are available online; however, they are too generic and lack proper design guidelines. Furthermore, the existing literature lacks real-time data analysis to generate timely, personalized recommendations through eCoaching. An appropriate eCoach-based personalized referral program can help people stay active and achieve their activity goals. There can be two types of goal types—short-term goals (e.g., weekly) and/or long-term goals (e.g., monthly). Achievement of the short-term goals (STG) contributes to the achievement of the long-term goals (LTG), and the LTG is the sum of the STG. Semantic rules in the ontology may enhance understandability in personalized recommendation generation. Most activity trackers, involving mobile apps and intelligent wearable devices (e.g., smart watches), predict future activity in terms of “steps” as a point prediction either with time-series forecasting, probabilistic approaches, or specific rules. However, point prediction is a very abstract concept. Therefore, in this context, a probabilistic interval prediction approach may be promising.

This study proposes a hybrid personalized recommendation generation concept in intuitive coaching with deep learning and ontology. We have developed an eCoaching prototype system that can perform a collection of activity data from actual participants with wearable activity sensors; process collected activity data with DL models to forecast step count; classify individual activity levels; calculate and compare activity intensity across different weeks with statistical methods; combine the results in an ontology for semantic knowledge representation and thereby generate personalized recommendations with SPARQL query engine against a rule base. The novel major contributions of this work include—(1) the design and development of an ontology model (OntoeCoach) for semantic representation of personal and personalized activity data, (2) proposing a novel algorithm that combines the OntoeCoach model with deep learning for hybrid recommendation generation with person based heuristic configuration, and (3) evaluation of the performance of time-series prediction, classification, and ontology models on both public (i.e., PMData) and private (i.e., MOX2-5 activity) datasets.

## Related work

We considered the overall activity eCoaching process in related work by classifying it into a data-driven approach and a rule-based approach. As eCoach design approaches and applications in eHealth are broader, therefore, included search results are mainly focused on technology-driven activity coaching for a healthy lifestyle and personalized feedback or recommendation generation.

**Table 1:** A comparison between our study and the related studies in a qualitative way.

Study	Hybrid recommendation	Semantic modeling with ontology and ontology tree in decision-making	Interval prediction	Observation with activity sensor	Incorporation of preference data	Logical recommendation generation
Our work	Yes	Yes	Yes	Yes	Yes	Yes
Dijkhuis et al.^22^	No	No	No	Yes	No	No
Hansel et al.^23^	No	No	No	Yes	No	No
Pessemier et al.^24^	Yes	No	No	Yes	Yes	No
Amorim et al.^25^	No	No	No	Yes	No	No
Oliveira et al.^26^	No	No	No	Yes	No	No
Petsani et al.^27^	No	No	No	No	No	No
Den et al.^28^	No	No	No	Yes	No	No
Chatterjee et al.^3^	No	Yes	No	No	No	No
Villalonga et al.^29^	No	Yes	No	No	No	No

### Data-driven approach

The literature search reveals that eCoach concepts with artificial intelligence (AI)-based tailored recommendation generation are still improving. Few studies have examined the use of actionable and data-driven predictive models^30^. Dijkhuis et al.^22^ analyzed personalized physical activity guidance for sedentary lifestyles using AI (ML and DL) algorithms at Hanze University. They collected daily step count data to train an AI classifier, estimated the likelihood of reaching an hourly step count goal, and then used a web-based coaching app to generate feedback. Hansel et al.^23^ designed and developed a fully automated web-based tutorial program. They used pedometer-based activity or step monitoring to increase their physical activity in a randomized group of patients with type 2 diabetes and abdominal obesity.

Pessemier et al.^24^ used raw accelerometer data for individual activity detection, accepted personal preferences to schedule activity recommendations, and generated personalized recommendations via tag-based and rule-based filtering. Amorim et al.^25^, and Oliveira et al.^26^ performed activity monitoring using a Fitbit over a randomized control trial study. They performed a statistical analysis to find the effectiveness of a multimodal physical activity intervention, including supervised exercise, fitness coaching, and activity monitoring of physical activity levels in patients with chronic nonspecific low back pain. Their research shows that physical activity is vital in managing chronic back pain. According to the review results, ML (e.g., Support Vector Machine (SVM), Decision Tree (DT), K-Nearest Neighbor (KNN), Principal Component Analysis (PCA), Linear Discriminator Analysis (LDA)) and DL (e.g., Multi-Layer Perceptron (MLP), Convolutional Neural Network (CNN), Recurrent Neural Network (RNN), Long-Term Short Memory (LSTM)) models have been used to classify, predict and generate recommendations in health settings^22–26,30–38^.

### Rule-based approach

Rule-based recommendation generation opens up new directions for eCoaching. Petsani et al.^27^ designed and developed an eCoach system for older adults to improve their adherence to physical activity. They followed electronic coaching guidelines set by a human therapist/physician or a trusted person chosen by the user who had access to stored health and wellness data and included or intervened in the coaching process. They concluded that health eCoaching is a complex process that requires careful planning and collaboration across many scientific fields, including psychology, computer science, and medicine. Braber et al.^28^ incorporated the eCoaching concepts into personalized diabetes management, where lifestyle data (e.g., food intake, physical activity, blood glucose values) were recorded and integrated into clinical rules to enable customized coaching for better lifestyle recommendations management. Chatterjee et al.^3^ focused on the design and development of a meaningful, context-specific ontology (“UiAeHo”) to capture unintuitive and raw insights from human-generated health data (e.g., sensors, interviews, questionnaires) using semantic models and unstructured observation metadata to create logical abstractions for rule-based health risk prediction in the eCoaching system. Villalonga et al.^29^ designed an ontology-based automated reasoning model to generate personalized motivational messages for activity guidance, taking into account behavioral traits. Therefore, ontologies can be a practical choice for rule-based decision-making with powerful design flexibility within the object-oriented design paradigm.

**Table 2:** A comparison between our previous studies and this extended study.

Study	Study focus	Dataset used	Recommendation type	Method focus
Chatterjee et al.^36^	Conceptualized the idea of weekly activity forecasting with statistical models and a rule-base for personalized rule-based recommendation generation in activity eCoaching	PMData	Personalized	ARIMA, SARIMA, Kalman Filter, Rule-database
Chatterjee et al.^37^	Conceptualized the idea of weekly activity forecasting and a rule-base for personalized recommendation generation with Ontology reasoning and querying in activity eCoaching	PMData	Personalized	LSTM, Ontology
Chatterjee et al.^38^	Semantic ontology model to annotate the machine learning (ML)-classification outcomes and personal preferences to conceptualize personalized recommendation generation with a hybrid approach in activity eCoaching with a focus on transfer learning approach to improve ML model training and its performance, and an incremental learning approach to handle daily growing data and fit them into the ML models (Support Vector, Naive Bayes, Decision Tree, K-Nearest Neighbour, Random Forest)	Zenodo Fitbit and MOX2-5	Personalized	Standard ML classification models, Ontology
Our work	Design and development of an extended ontology model for semantic representation of personal and personalized activity data, and algorithm development to include time-series forecasting, time-series physical activity level classification, and statistical metrics in the ontology model for hybrid recommendation generation with person-based heuristic configuration and the verification of the algorithm against different datasets with existing and derived metrics	PMData and MOX2-5	Personalized	Deep learning models, Ontology, Probabilistic Interval Prediction, Statistical Metrics

In state-of-the-art research, the feasibility analysis of DL time-series classifiers and prediction models in physical activity detection is demonstrated to design an ML or DL pipeline. However, this study shows its application one step ahead by applying DL models, statistical methods, and OWL ontology in real-time activity guidance to improve sedentary lifestyles through goal management skills. In particular, this study has utilized the ML and DL concepts in the followings objectives $^\smallsmile$ (1) an MLP model to classify individual daily physical activity into multiple levels such as sedentary, low physically active (LPA), medium physically active (MPA), and vigorous physically active (VPA), (2) a CNN1D model for univariate “step” forecasting, (3) state-of-the-art statistical methods to calculate weekly activity intensity, (4) mapping the time-series point prediction to an interval prediction, and (5) the creation of an OWL ontology for semantic modeling of personal preferences, activity predictions, and the generation of personalized recommendations with SPARQL against a rule base.

To verify the above objectives, we use sensor data processed by Fitbit Versa and MOX2-5 wearable activity sensors instead of raw signal data (e.g., accelerometer, gyroscope) for personal activity prediction and classification. Moreover, to explain the study’s relevance, we proposed an algorithm to annotate the activity prediction outcomes in an ontology for personalized recommendation generation. Semantic annotation can more easily identify causal relationships between data inputs and recommendation results. The above-mentioned study by Pessemier et al. focused on recommendation generation at the “Community” level whereas this work targets activity coaching and recommendation generation at the “Personal” level. To the best of our knowledge, no similar work has been published or made available online, therefore, instead of a quantitative evaluation, a qualitative comparison between our study and the related activity coaching studies has been described in Table  1. Our present study is the extended version of our previous studies^36–38^. In Table 2, we elaborated on the novelty of this study and how this study differs from our previous studies and added more value, with a qualitative comparison.

## Proposed hybrid recommendation generation

In this section, we begin by defining and explaining the OntoeCoach ontology proposed in our research. We then delve into the problem formulation and algorithm. Finally, we conclude this section by presenting the derived time complexity of the proposed model.

### Ontology modelling

The proposed OntoeCoach ontology follows the following knowledge representation phases—abstraction or dictionary (*L*) of mapping rules, abduction phase (*B*) of hypothesis generation rules, deduction (*C*), and induction of operator reduction rules for generalization (*D*). The generated recommendation spanning tree (*T*) follows a binary structure, and the syntactic knowledge representation of *T* helps to solve the understandability problem when generating personalized lifestyle recommendations.

Our proposed OntoeCoach ontology is a tree-like hierarchical structure ($O_h$) with the following properties. Formally, the ontology (*O*) may be represented as $\Omega$ = {*C*, *R*}, where *C* is the concept set and *R* is a relation set. The total levels in an ontology hierarchy is represented by *H* = Levels ($O_h$), 0 $\le$ *n* $\le$ *H*, where *n* $\in$ $Z^+$, *n* = 0 and represents the root node. When a model is classifying (*O*) at a level *n*, can be denoted as $C_{n,i}$, where *i* $\in$ {0, 1, ...| $C_n$ |}. | *C* | is number of instances classified as class *C*. The edge between node $C_{n,i}$ and its parent node $C_{(n-1, j)}$ is defined as *E* = Edge ($C_{n,i}$, $C_{(n-1, j)}$). We re-used the concept and extended our ontology representation with the following four tuples:

1 $$\begin{aligned} O = \{{O_a, R, I, P}\}, \end{aligned}$$

where $O_a$ is defined as $O_a$ = {$O_{a1}$, $O_{a2} \ldots O_{an}$}, it represents “*n*” concepts or classes and each $O_{ai}$ has a set of “*j*” attributes or properties $\forall$ $P_i$ = {*p*1, $p2 \ldots pi$} where *n*, *i*, *j* $\in$ $Z^+$. We denote a set of binary relations between the elements of $O_a$ by *R*. *R* holds two subsets *H* for the inheritance relationship among concepts and *S* for the semantic relationship between concepts with a domain and range. We represent a knowledge base with a set of object instances by *I*. *P* represents a set of axioms to model *O* and it includes domain-specific constraints to model an Ontology with $O_a$, *R*, and *I*. The knowledge (*K*) in the ontology has been expressed with two tuples, defined as:

2 $$\begin{aligned} O = \{{Onto_{ActivityReco}, Rules_{ActivityReco}}\}, \end{aligned}$$

where the components of $Onto_{ActivityReco}$ and $Rules_{ActivityReco}$ are defined as:

3 $$\begin{aligned}{} & {} Onto_{ActivityReco} = \{{OA_L, OA_B, OA_C, OA_D}\}, \end{aligned}$$

4 $$\begin{aligned}{} & {} Rules_{ActivityReco} = \{{RA_L, RA_B, RA_C, RA_D}\}, \end{aligned}$$

where $OA_L$, $OA_B$, $OA_C$, $OA_D$ are the knowledge bases, consisting of lexicon, abduction, deduction, and induction phases for personalized physical activity recommendation. On the contrary, $RA_L$, $RA_B$, $RA_C$, $RA_D$ are rule sets to match with the abstraction, abduction, deduction, and induction interfaces, respectively. $OA_B$, $OA_C$, $OA_D$ are representations of properties *P* of concepts $O_a$, data or entities (e.g., activity variables), and they follow a simple representation of *P*(*X*|*Y*) or *P*(*Y*|*X*) based on the relational mapping, where, *P* is attributes or properties in *O*, and *X*, *Y* are components of activity variables.

**Figure 1:**
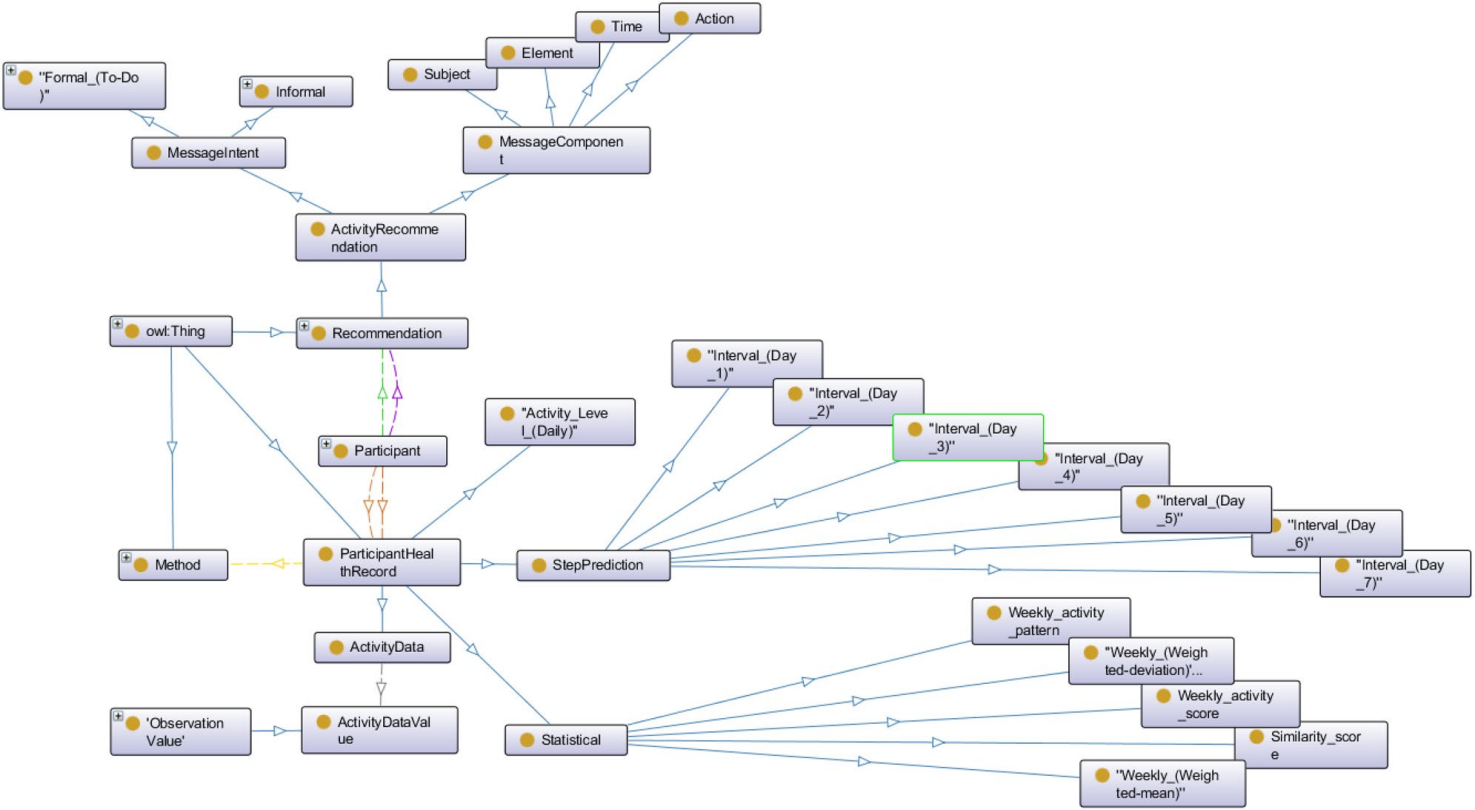
The high-level structure of the proposed OntoeCoach Ontology.

Rule sets help to explain the logic behind recommendation generation. All rule execution internally follows a binary tree structure, where non-leaf nodes contain semantic rules to be executed ($A| A \rightarrow B$), and leaf nodes have results (*B* or recommended message). The edges contain decision statements (true or false). For interactively navigating the relationships of our OWL ontology, we implemented the high-level structure of OntoeCoach ontology (see Fig.  1) in OntoGraf using the Protege. The key object properties, domain, range, property, and cardinality of OntoeCoach ontology are described in Table 3. The OntoeCoach ontology is the extended version of our previous ontological studies as elaborated in^13,38^ and annotates the subsequent data types for reasoning—sensor observation (e.g., activity sensor), personal information, and personal preference data, personalized recommendations, and participant health records (e.g., activity level, step prediction, statistical metrics) in the processed forms. The ontology metrics used in our OntoeCoach design are—(a) Metrics (Axiom (n = 965), Logical axiom count (n = 327), Declaration axiom count (n = 310), Class count (n = 90), Object property count (n = 81), Data property count (n = 128) and Annotation property count (n = 13)), (b) Class axioms (SubClassOf (n = 167), EquivalentClasses (n = 12), Hidden GCI Count (n = 12)), (c) Object property axioms (SubObjectPropertyOf (n = 30), InverseObjectProperties (n = 8), ObjectPropertyDomain (n = 8), ObjectPropertyRange (n = 8), and SubPropertyChainOf (n = 2)), (d) Data property axioms (SubDataPropertyOf (n = 9), DataPropertyDomain (n = 25), and DataPropertyRange (n = 25)), and Annotation axioms (AnnotationAssertion (n = 328)). “n” signifies counts $\ge$ 0.

**Table 3:** Key object properties, domain, range, and cardinalities of the ontoeCoach ontology.

Object properties	Domain	Range	Cardinality
HasPersonalHealthRecord	Participant	HealthRecord	Some
HasPersonalDataInfo	Participant	PersonalData	Some
HasPersonalPreferences	Participant	Preferences	Some
HasReceivedPersonal Recommendation	Participant	Recommendation	Some
HasHealthStatus	Participant	ParticipantStatus	Some
HasbeenCollectedBy	ActivityData	ActivityDataValue	Some
HasTimeStamp	ActivityDataValue, Questionnaire, Recommendation, ParticipantHealthRecord	TemporalEntity	Some
Has Measurement Capability	ActivityDevice	Measurement Capability	Only
HasOutput	ActivityDevice	Sensor Output	Some
Observes	ActivityDevice	Property	Only
Detects	ActivityDevice	Stimulus	Only
Feature of interest	Observation	Feature of Interest	Only
Observation result	Observation	Sensor Output	Only
ObservedBy	Observation	Sensor	Only
Is property of	Property	Feature of Interest	Some
HasProperty	Feature of Interest	Property	Some
HasIntervalDay	Participant	StepPrediction	Some
HasActivityLevel	Participant	Activity_Level_(Daily)	Some
HasStatValue	Participant	Statistical	Some

### Problem formulation and proposed algorithm

In this study, the recommendations are generated to maximize weekly individual physical activity levels and to minimize sedentary time. The maximization problem focuses on maintaining a moderate activity level for an entire week (i.e., $\sum$ Days $\in$ (1, 2...n) $\forall$ $n=7$. We consider multiple expression for the activity maximization problem. We maximize the four parameters – 1) $\sum$ Moderate$_{Activitytime}$ > 150, 2) $\sum$ GoalScore$_{daily}$ $\ge$ 21, 3) 0 $\le$ $\sum$ $\mu _{S}$ $\le$ 32, and 4) SimilarityScore$_{weekly}$ $\ge$ 0. These parameters are maximized subject to the multiple conditions such as—(1) Moderate$_{Activitytime}$ $\ge$ 21.45, (2) GoalScore$_{daily}$ $\ge$ 3, (3) 0 $\le$ PerformanceScore$_{daily}$ $\le$ 32, (4) $C_V$ $\rightarrow$ *P*, (5) *P* $\rightarrow$ *R*, (6) $\sum$ *P* = 1, and (7) ModerateActivitytime = 2 * VigorousActivitytime.

**Table 4:** The “Activity Level” classification rules following the WHO guidelines.

Level (score)	Rule(s)$^{\textbf {a}}$
Sedentary (0)	((step < 5000) $\wedge$ (VPA*2 + MPA) *7 < 90 $\wedge$ LPA $\ge$ 0)) $\vee$ (step < 5000)
Low physical active (1)	((step > 4999) $\wedge$ (VPA*2 + MPA) *7 $\ge$ 90 $\wedge$ (VPA*2 + MPA) *7 < 210) $\vee$ (step > 4999 $\wedge$ step < 7500)
Active (2)	((step > 4999) $\wedge$ (VPA*2 + MPA) *7 $\ge$ 210 $\wedge$ (VPA*2 + MPA) *7 < 300) $\vee$ (step > 7499 $\wedge$ step < 10,000)
Medium physical active (3)	((step > 4999) $\wedge$ (VPA*2 + MPA) *7 $\ge$ 300 $\wedge$ (VPA*2 + MPA) *7 < 360)) $\vee$ (step > 9999 $\wedge$ step < 12,500)
High physical active (4)	((step > 4999) $\wedge$ (VPA*2 + MPA) *7 $\ge$ 360) $\vee$ (step > 12,499)

$$^a MPA$$ = 2 *VPA*.

**Table 5:** In context recommendation conditions and corresponding rules (Rule-base) for test set-up.

No.	Semantic rule(s) (R) and condition
1	(hasActivityLevel == 0) IMPLIES (Sedentary AND hasPhysicalActivityLevel) (hasActivityLevel == 1) IMPLIES (Low_physically_active AND hasPhysicalActivityLevel) (hasActivityLevel == 2) IMPLIES (Physically_active AND hasPhysicalActivityLevel) (hasActivityLevel == 3) IMPLIES (Moderate_physically_active AND hasPhysicalActivityLevel) (hasActivityLevel == 4) IMPLIES (Vigorous_physically_active AND hasPhysicalActivityLevel)
2	((hasSedentaryBouts - daily_sedentary_goal_time as set in goal) > 0) IMPLIES (Sedentary_hour_negative) ((hasSedentaryBouts - daily_sedentary_goal_time as set in goal) <= 0) IMPLIES (Sedentary_hour_positive)
3	((hasSteps - daily_step_goal as set in goal) => 0) IMPLIES (Steps_positive) ((hasSteps - daily_step_goal as set in goal) < 0) IMPLIES (Steps_negative)
4	((hasMPAMinutes - daily_MPA_goal as set in goal) OR (hasVPAMinutes*2 - daily_VPA_goal as set in goal) => 0) IMPLIES (Activity_minute_positive) ((hasMPAMinutes - daily_MPA_goal as set in goal) OR (hasVPAMinutes*2 - daily_VPA_goal as set in goal) < 0) IMPLIES (Activity_minute_negative)
5	((hasWeeklyStepPrediction - weekly_step_goal as set in goal) => 0) IMPLIES (Step_forecast_trend_postive) (hasWeeklyStepPrediction - weekly_step_goal as set in goal < 0) IMPLIES (Step_forecast_trend_negative)
6	((hasSteps - daily_step_goal as set in goal) => 0) AND ((hasMPAMinutes - daily_MPA_goal as set in goal) OR (hasVPAMinutes*2 - daily_VPA_goal as set in goal) => 0) AND (hasTotalSleepTime => (daily_sleep_goal as set in goal *60)) AND ((hasSedentaryBouts - daily_sedentary_goal_time as set in goal) <= 0) IMPLIES (Daily_Goal_achieved)
7	(hasCurrentWeeklyDeviation > hasPreviousWeeklyDeviation) AND (hasSimilarityScore > 0) IMPLIES (Weekly_performance_deviation_trend_negative)
8	(hasCurrentWeeklyDeviation <= hasPreviousWeeklyDeviation) AND (hasSimilarityScore == 0) IMPLIES (Weekly_performance_deviation_trend_positive)
9	((hasSteps - weekly_step_goal as set in goal) => 0) AND ((hasMPAMinutes - weekly_MPA_goal as set in goal) OR (hasVPAMinutes*2 - weekly_VPA_goal as set in goal) => 0) AND (hasTotalSleepTime => (weekly_sleep_goal as set in goal *60)) AND ((hasSedentaryBouts - weekly_sedentary_goal_time as set in goal) <= 0) IMPLIES (Weekly_Goal_achieved)
10	(Sedentary + Low_physically_active + Moderate_physically_active + Vigorous_physically_active + Sedentary_hour_negative + Sedentary_hour_positive + Steps_negative + Steps_positive + Activity_minute_negative + Activity_minute_positive + Step_forecast_trend_postive + Step_forecast_trend_negative + Daily_Goal_achieved + Daily_Goal_not_achieved + Weekly_Goal_achieved + Weekly_Goal_not_achieved + Good_weather + Bad_weather + Weekly_performance_deviation_trend_positive + Weekly_performance_deviation_trend_negative = 1)

Activity goals can be system-defined (i.e., generic goals defined by WHO) or user-defined, as athletes may have different goal plans than ordinary people. According to the World Health Organization, adults (ages 18–64) should complete at least 150–300 min (2.5–5 h) of moderate-intensity aerobic activity (MPA); or at least 75–150 minutes of vigorous aerobic activity (VPA) or equivalent moderate- and vigorous-intensity exercise to stay active. We have added the daily activity scores to calculate each week’s individual goal achievement scores (see Table  4). In Table  4, the right column represents the standard rules to determine the activity level on a daily basis. The left column represents the type of activity level and their numeric representation as a daily score value. Activity eCoach is designed to maximize target scores through continuous activity monitoring and personalized recommendation generation.

For validation, we used rule-based personalized activity recommendation generation and *SPARQL* queries to motivate eCoach participants to stay active by reducing their sedentary time. Ontologies annotate recommendation messages to describe their attributes, metadata, and content information outside the static text form. Recommendation messages can be both formal and informal. Additionally, the rule base helps explain the logic behind recommendation generation through logical *AND*, *OR*, and *NOT* operations.

In this work, the *SROIQ* description logic is used as the formal argument logic (see Table 5). The Table  6 contains a defined set of recommended messages for OntoeCoach ontology validation based on the used dataset. For each condition described in Table  5, the *RG* module runs a *SPARQL* query to determine the type of referral message sent to the individual daily. This study grouped eight semantic rules into activity-level categories (9) and satisfiability categories (1). The integrated concepts and rules are easy to follow and apply. Custom recommendations are generated using the structure [(rule) IMPLIES (suggestion variable) $\rightarrow$ recommendation message]. In Table  5, the semantic rules have been created to define relationships and constraints between different entities or concepts within the activity eCoach knowledge representation system. These rules help capture the data’s meaning and semantics and enable reasoning and inference capabilities. Here are the steps involved in defining the semantic rules—(a) *Identify the Entities*: We identified the entities and concepts for which we want to define semantic rules. These entities represent objects, properties, and relationships in the physical activity domain. (**b**) *Define the Relationships*: We specified the relationships between the entities which includes identifying the type of relationship (e.g., “is-a,” “part-of,” “has-property”) and the directionality of the relationship. (**c**) *Define Constraints*: We determined constraints or conditions that need to be satisfied for the relationships to hold true. These constraints involve logical operations, comparisons, or other specific criteria. (**d**) *Rule Representation Format*: We selected a suitable format or language to represent the semantic rules. Our common formats include formal languages, such as OWL (Web Ontology Language) or RDF (Resource Description Framework), and rule-based languages, such as SPARQL (SPARQL Protocol and RDF Query Language). (**e**) *Expression of the Rules*: We expressed the semantic rules using the chosen representation format. This involves writing the rules based on the identified entities, relationships, and constraints. The syntax and semantics of the chosen format will guide the rule expression. (**f**) *Validate and Test the Rules*: We validated the semantic rules to ensure their correctness and consistency. We planned to test the rules against sample data or scenarios to verify their behavior and evaluate their effectiveness. (**g**) *Refine and Iterate*: We refined the rules based on feedback, domain expertise, or real-world use cases. We iterated the process of rule creation, testing, and refinement to improve the quality and accuracy of the semantic rules. Overall, the creation of semantic rules required a good understanding of the domain, the entities involved, and the desired semantics. Collaboration with domain experts and leveraging existing ontologies or knowledge bases had also been valuable in the rule-creation process.

Measurable parameters related to the activity of a particular participant in a timestamp are obtained at preference-based intervals based on SPARQL queries. Rules (1–9) in Table  5 assign Boolean values to variables, ensuring consistency. We have verified using Ontology Reasoner that the correct recommendation message is triggered for a particular situation. However, ensuring that no variable patterns would make the entire rule unsatisfactory is essential. We’ve made sure that only one message is active at a time. Here we have a formal guarantee that neither two “once a day” messages can be active at the same time, nor can there be a model with a reasoner output each time for every possible combination of variables.

Let us consider a case, if we put the different variables used in the nine rules as described in Table 5 to generate respective propositional variables (see Table 6). In that case, we will have an exponential number of possible participants. A traditional way to ensure the presence of a model negates all our rules and provides the same. Therefore, this formula is not satisfactory. Since two messages cannot be triggered simultaneously, we added a rule (Rule-10) to meet the exact requirement, and the variables used in the suggestion start once a day. If (rule-10) is false, the entire rule set (considered significant conjunction) is set to false, then there is no model as output, and we can debug our rules if needed. When set to true, we have a formal guarantee that no two “once a day” messages will fire simultaneously, regardless of the true value we feed into the rule base. All rule execution internally follows a binary tree (BT) structure, where the non-leaf nodes contain the semantic rules to be executed (*A* | *A* $\rightarrow$ *B*), and the leaf nodes have the results (B or recommendation message). Edges have decision statements (true or false). In this way, satisfiability and understandability (or explainability) issues are addressed in custom recommendation generation in our Activity eCoach system. The proposed personalized hybrid recommendation generation approach is described in Algorithm 1.

**Table 6:** Propositional variables and corresponding recommendation messages.

Type	Propositional variable (P)	Description
A-1	Sedentary	Please continue a light activity (e.g., sports 1–3 days/week, a walking goal of 5000 to 7499 steps/day)
A-2	Low_physically_active	Please continue more activity (e.g., sports 3–5 days/week, a walking goal of 7500–9999 steps/day) OR do a minimum 150–300 min (2.5–5.0 h) of moderate-intensity aerobic exercise or minimum 75–150 min of high-intensity aerobic exercise or do an equivalent combination of moderate and high-intensity activities in a week to stay physically active
A-3	Physically_active	Please continue the same or more activities based on your goal (e.g., sports 3–5 days/week, a walking goal of 7500 to 9000 steps/ day)
A-4	Moderate_physically_active	Please continue the same or more activities based on your goal (e.g., sports 3–5 days/week, a walking goal of 10,000 to 12,499 steps/ day)
A-5	Vigorous_physically_active	Please continue the same or more activities based on your goal (e.g., sports 5+ days/week, a walking goal of 12,500+ steps/day)
A-6	Sedentary_hour_negative	Please be active for z h. more as today you were z h. more sedentary beyond your goal
A-7	Sedentary_hour_positive	You were very active today and z hr. less sedentary; therefore, you can take that h. of rest tomorrow
A-8	Steps_negative	Please continue x steps more tomorrow to achieve your weekly goal of x1 steps
A-9	Steps_positive	You have performed extra x steps today beyond your goal; therefore, you can do x steps less tomorrow or you can carry out the same pace. You are x1 step behind to achieve your weekly goal (OR) congratulations! You have achieved your weekly target
A-10	Activity_minute_negative	Please continue more activity of n min tomorrow to achieve n1 min of a weekly goal
A-11	Activity_minute_positive	You have performed extra m minutes of activity today beyond your goal; therefore, you can be m mins. of less highly active tomorrow or you can carry out the same pace. You are n1 mins. behind to achieve your weekly goal (OR) congratulations! You have achieved your weekly target
A-12	Step_forecast_trend_postive	Based on your weekly step forecast trend in this Week-N you can achieve the step goal
A-13	Step_forecast_trend_negative	Based on your weekly step forecast trend in this Week-N you cannot achieve the step goal. On Week-XX and Week-XY weeks, you were very active. Please try to follow similar activity patterns
A-14	Daily_Goal_achieved	Good work. Please keep it up tomorrow. You are active and completed the goal for today. Overview: You have performed X steps today. You slept Y h. You were sedentary for Z h. You were M min medium active. You were N min highly active
A-15	Daily_Goal_not_achieved	You must improve to meet the daily goal. Please stay active tomorrow. Overview: You have performed X steps today. You slept Y h. You were sedentary for Z h. You were M min medium active. You were N minutes highly active
A-16	Weekly_performance_deviation _trend_positive	Congratulations! You have maintained a good weekly activity pattern
A-17	Weekly_performance_deviation _trend_negative	Your weekly activity pattern must be improved
A-18	Weekly_Goal_achieved	Good work. Please keep it up next week. You are active and completed the goal for this week
A-19	Weekly_Goal_not_achieved	You must improve to meet the weekly goal. Please stay active next week and try to overcome the shortcomings of this week. On Week-XX and Week-XY weeks, you were very active. Please try to follow similar activity patterns


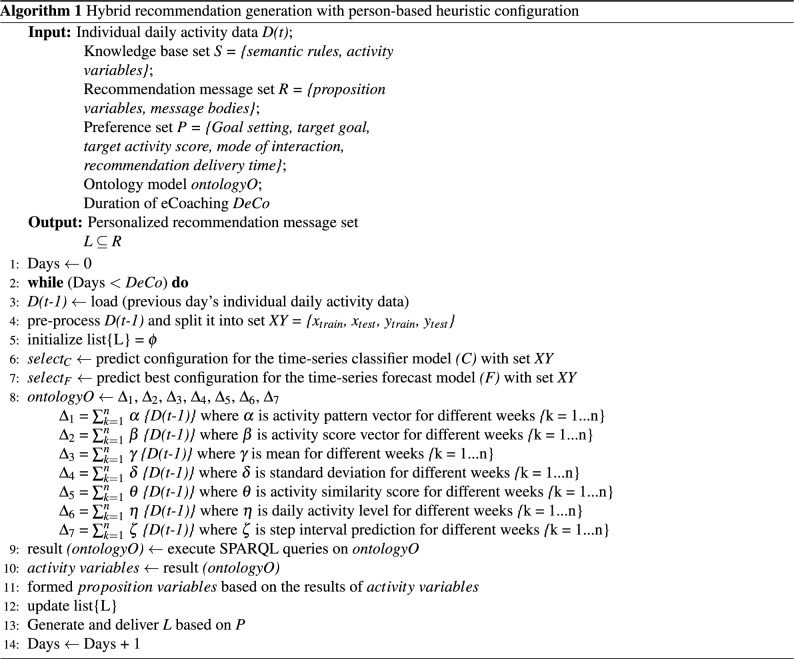


To assess the performance of Algorithm 1 more effectively, we consider its time complexity^39^. This analysis helps to understand how the algorithm’s effectiveness scales with increasing input size. The time complexity is typically expressed using big O notation, which provides the maximum growth rate of the algorithm’s execution time. By analyzing time complexity, we can estimate the efficiency and scalability of the algorithm, compare the performance of different algorithms, and identify any design bottlenecks. In the case of our proposed algorithm, the time complexity is quadratic, denoted as $O(n^2)$, due to the presence of a nested loop. Here, “n” represents the input size, with a value greater than 0. This quadratic time complexity indicates that the running time of the algorithm grows quadratically with the input size.

## Activity eCoach system overview

**Figure 2:**
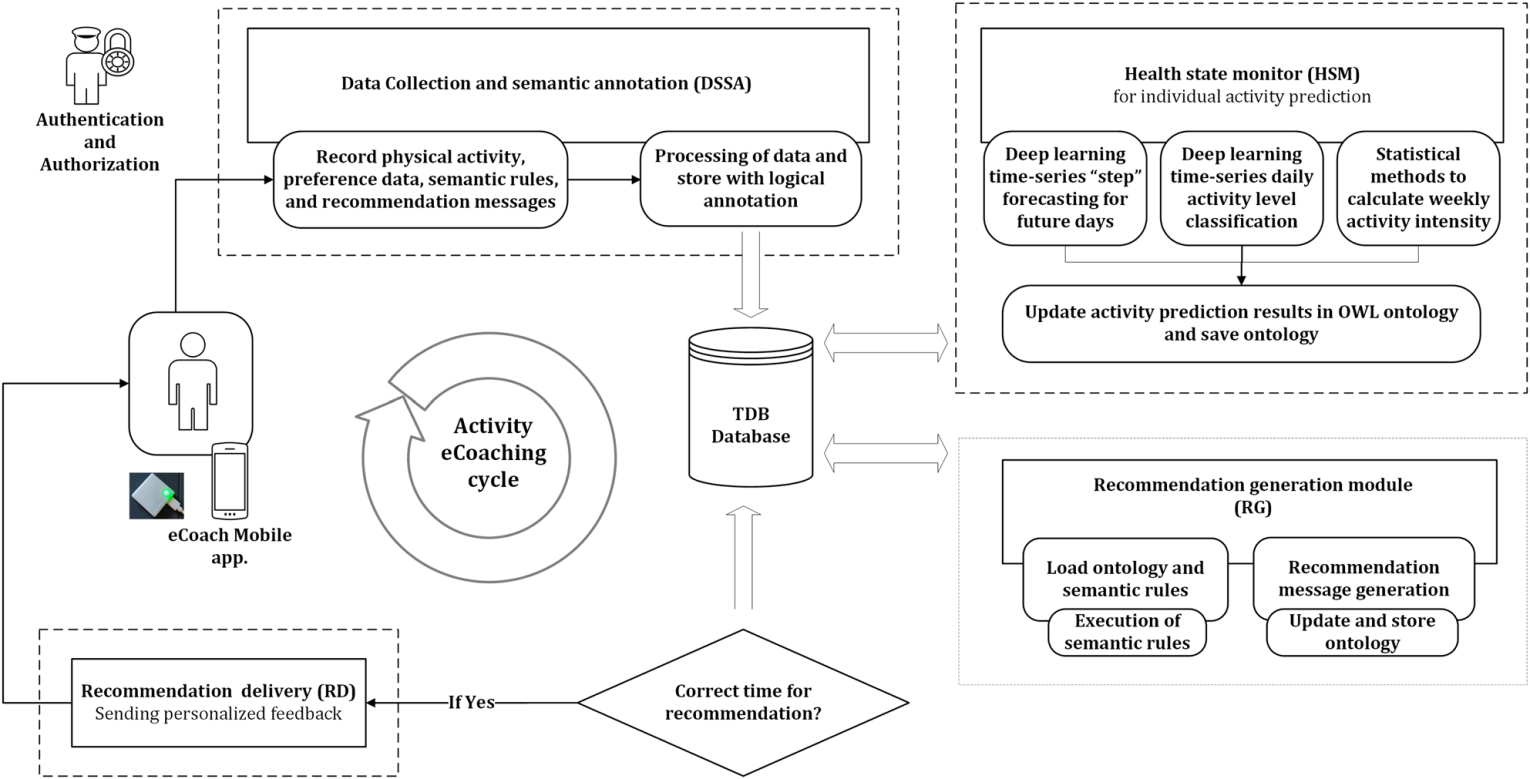
The data flow in the Activity eCoach system includes all components and their connections. In this, TDB represents a tuple database.

This section describes a model for activity eCoaching. We followed an iterative and incremental approach to design and develop our Activity eCoach that follows a modular design with four primary modules—(1) data collection and semantic annotation (DSSA), (2) health state monitoring (HSM), (3) recommendation generation (RG), and (4) recommendation delivery (RD). The data flow in the activity eCoach prototype system is depicted in Fig. 2.

After collecting personal, person-generated activity and preference data, the DSSA module stores them in a tuple database (TDB) using semantic annotation. Moreover, the DSSA module records pre-defined rulesets and recommendation messages set to be generated as a part of personalized recommendation generation and store them in the database. The rules and recommendation messages can be updated based on the context. We plan to use a standard wearable CE-approved activity sensor (e.g., MOX2-5) for activity data collection. Furthermore, we prepared a set of questionnaires to collect personal preference data for recommendation planning. Personal preference data includes goal settings (such as daily, weekly, or monthly), target goals (such as moderately active or vigorously active), goal scores, interaction types, or recommendation delivery (such as text, audio, or graphics), and the recommended delivery time. Participants can review and update their preference information at any time.

The HSM module consists of the following three submodules—classification, forecasting, and statistical analysis (SA). The classification submodule classifies daily time-series activity data into the following activity levels: sedentary (0), LPA (1), MPA (2), and VPA (3) (see Table 3). The prediction submodule is responsible for forecasting daily steps for the next 7-days based on the temporal pattern in individual step data. The SA submodule calculates the weighted mean, activity pattern, and similarity score between the weekly achieved activity score and weekly goal score to understand the weekly activity intensity. All the outcomes of the DP module are semantically annotated in OntoeCoach ontology and followed by stored in the TDB. Furthermore, we designed a pipeline to automate the process. An incremental approach helped to keep the DL models updated with real-time, growing activity data.

The RG module runs a scheduler periodically to query and process individual activity prediction results from the TDB database with a SPARQL query engine and a KB. In KB, all the semantic rules are stored for recommendation generation. Some suggestions should be made to the participants of the semantic data source if some specific variables are inferred to be true. Semantic rules consist of propositional variables using (IMPLIES), (OR), (AND), and (NOT) operations. RG modules trigger logical structure rules (A IMPLIES B) or in a logically identical way (NOT(A) or B). Following, individual recommendation data are updated in the OntoeCoach ontology against a timestamp and stored in the TDB. The RD module periodically accesses TDB for personal preference data and generates individual recommendation data to send personalized feedback based on personal preferences. Additionally, it meaningfully displays a reflection of ongoing activity through continuous and discrete personal health data, notifications, and recommended messages.

All the modules follow a microservice architecture. The exposed eCoach interfaces are protected with multifactor authentication and authorization (OAuth2) to allow legitimate users only^40–42^. The DC, RG, and RD modules are written in Java (JDK 11+) programming language with SpringBoot Framework. The HSM module is written in Python (V. 3.8.x) programming language with Flask Framework, and Python DL libraries, such as sktime, and Keras. Open-source Apache libraries (such as Jena, Jena Fuseki, and Tomcat 9.x) have been used for ontology implementation and eCoach service deployment.

## Materials and methods

This section describes materials and methods that are utilized to run the overall experiment.

### Experimental setup

We used Python 3.8.5-supported language libraries such as pandas (v. 1.1.3), NumPy (v. 1.21.2), SciPy (v. 1.5.2), Matplotlib (v. 3.3.2), Seaborn ( v. 0.11.0), Plotly (v. 5.2.1), scikit-learn or sklearn (v. 0.24.2), Keras (v. 2.6.0), and Graph Viz (v. 2.49.1) to process data, build and train deep learning models. We set up a Python environment on a Windows 10 operating system using the Anaconda distribution and installed Jupyter Notebook v. 6.4.5 for development, model analysis, and data visualization. The target system consists of 16 GB RAM and 64-bit architecture. Due to the small size of the dataset, we used the CPU to run the experiments.

### Data collection

We followed ethical guidelines during the collection, processing, and representation of personal and personalized activity data in our activity eCoach prototype system. We focused collection of activity data only for adults (aged 18–64). The bodybuilders, pregnant women, and persons with a severe medical history and chronic illness were excluded from the study. This work includes the following two data sets.

#### PMData public datasets

We used the anonymized PMData public physical activity dataset of *n* = 15 adult *(male 12; female 3)* for model training and testing. The activity dataset was collected from a Fitbit Versa 2 fitness smartwatch to PMSys sports logging smartphone application^43^. We received nearly 114–152 days of recordings from each participant, for a total volume of 2244 recordings. This dataset shows several features related to physical activity, e.g., VPA). However, we chose the “steps” metadata file and excluded sleep-related features since sleep tracking is out of scope. We excluded activity data for participant P_12 from the analysis due to a lack of LPA information.

#### MOX2-5 real-time datasets

We collected 30–45 days of physical activity data from *n* = 16 adults *(male 12; female 4)* in Grimstad, Norway anonymously, using the wearable activity sensor MOX2-5 (CE certified)^44^. We followed Norwegian ethical guidelines to collect real-time activity data from actual participants with signed consent forms. It produced 539 volume records. With the permission of the Norwegian Study Data Center (NSD), we collected and evaluated the personal data of the participants in this study following data protection law. The characteristics of the participants are recorded in Table 7. Table 8 describes the features of the MOX2-5 dataset.

**Table 7:** Participant characteristics (N = 16).

Factors	Mean ($\mu$)	SD ($\sigma$)	Min	Max	$P_{25}$	$P_{50}$	$P_{75}$
Age	35.375	± 7.03	21	51	30.8	35.5	39.0
Height (cm)	173.5	± 8.02	158.5	184.0	167.6	173.3	180.5
Weight (kg)	77.0	± 16.36	55.0	107.0	65.0	72.0	90.5
BMI	25.38	± 3.93	19.41	31.604	22.0	25.8	27.9
Duration (days)	33.6875	± 5.41	30	48	30.6	31.0	34.3
Total sedentary seconds	24,49,171	± 1,051,610.5	590,028	4,261,190	–	–	–
Total VPA seconds	41,887.81	± 60,688.5	112	256,896	–	–	–
Total MPA seconds	53,231.75	± 17,965	23,402	95,730	–	–	–
Total LPA seconds	154,647.1	± 66,540.6	32,272	254,332	–	–	–
Total steps	366,703.3	± 87,202.25	52,551	588,132	–	–	–

**Table 8:** Attributes of the MOX2-5 datasets.

Attributes	Type	Description
Date	String	Recorded activity date
Time	String	Recorded activity time
UploadStatus	Character	Indicates uploading status: ‘H’ and ‘L’
IMA	Integer	Total activity intensity
WeightBearing	Integer	Total weightbearing seconds
Sedentary	Integer	Total sedentary seconds
Standing	Integer	Total standing seconds
LPA	Integer	Total low physical activities seconds
MPA	Integer	Total moderate physical activities seconds
VPA	Integer	Total vigorous physical activities seconds
Steps	Integer	Total daily step count

**Table 9:** The relation between activity intensity (IMA) and activity type.

Activity type	Rule
LPA	0 $\le$ Activity intensity (IMA) $\le$ 400
MPA	401 $\le$ Activity intensity (IMA) $\le$ 800
VPA	Activity intensity (IMA) $\ge$ 801

### Feature selection

Activity data shows steps per minute. Therefore, we turned it into a daily step count for daily step count prediction. We used the Augmented Dicky-Fuller (ADF) hypothesis test^45^ with Autolog = “AIC” and Regression = “CT/C” to verify the stationarity of the time series data. We used seasonal decomposition to analyze the data’s trend, seasonal and residual components. We transformed non-stationary data into stationary using the differential transformation method. It helped to remove trends and seasonality in time series data. We observed the lag values (X-axis) and correlations (Y-axis) using the 2D autocorrelation (ACF) plots and partial autocorrelation (PCF) with finite lag values (e.g., 25, 50) to plot observations. ACF and PCF have been useful for parameter selection in time series forecasting models. Additionally, we used the forward and backward filling methods to handle missing data.

The relevant features obtained from the MOX2-5 sensor are − time stamp, the intensity of activity (IMA), seconds sitting, seconds bearing weight, seconds standing, seconds LPA, seconds MPA, seconds VPA, and steps per minute. “Step” and “IMA” are the most valuable and robust features of the sensor-based MOX2-5 dataset since other attributes (except timestamp) are almost derived (e.g., LPA, MPA, and VPA are defined as IMA derivative of Table  9). IMA has a strong relationship with step count and is primarily used as a measure of activity. For MOX2-5 sensors, sedentary time is the period without physical activity, including leisure and sleep. The relationship between sitting and active (LPA/MPA/VPA) time can be written as $\Sigma$(sitting, active, weight-bearing, standing) = 60 s. Activity intensity values can be correlated to energy expenditure expressed in metabolic values (METs). It allows the following classification—LPA: 1.5 to 3.0 METS, MPA: 3.0 to 6.0 METS, and VPA: 6.0 or more METS.

The Shapiro-Wilk normality test method^2^ uncovered that the individual data sample and their columns did not look like a Gaussian distribution. Normality testing is a hypothesis testing method using P-value > $\alpha$ = 0.05 (i.e., the sample looks like a Gaussian distribution) and P-value < $\alpha$ = 0.05 (i.e., the sample does not look Gaussian)^2^. The $\alpha$ indicates the confidence interval. For feature selection, we used Spearman’s correlation analysis, which reveals the strength of the linear relationship between features according to the value of the correlation coefficient (r)^2^. We removed functions that strongly depend on the value |*r*| > 0.72. SelectKBest using chi-square, ExtraTreesClassifier, and Principal Component Analysis (PCA) facilitates feature ranking and feature selection in two datasets^4,46,47^. PCA uses the variance ratio of the eigenvalues of the eigenvectors to the total eigenvalues. The selected temporal activity data are continuous for both datasets. We eliminated participant data that is less than a month old, redundant, noisy, incomplete, or missing. For prediction, we considered univariate daily steps from two datasets.

**Figure 3:**
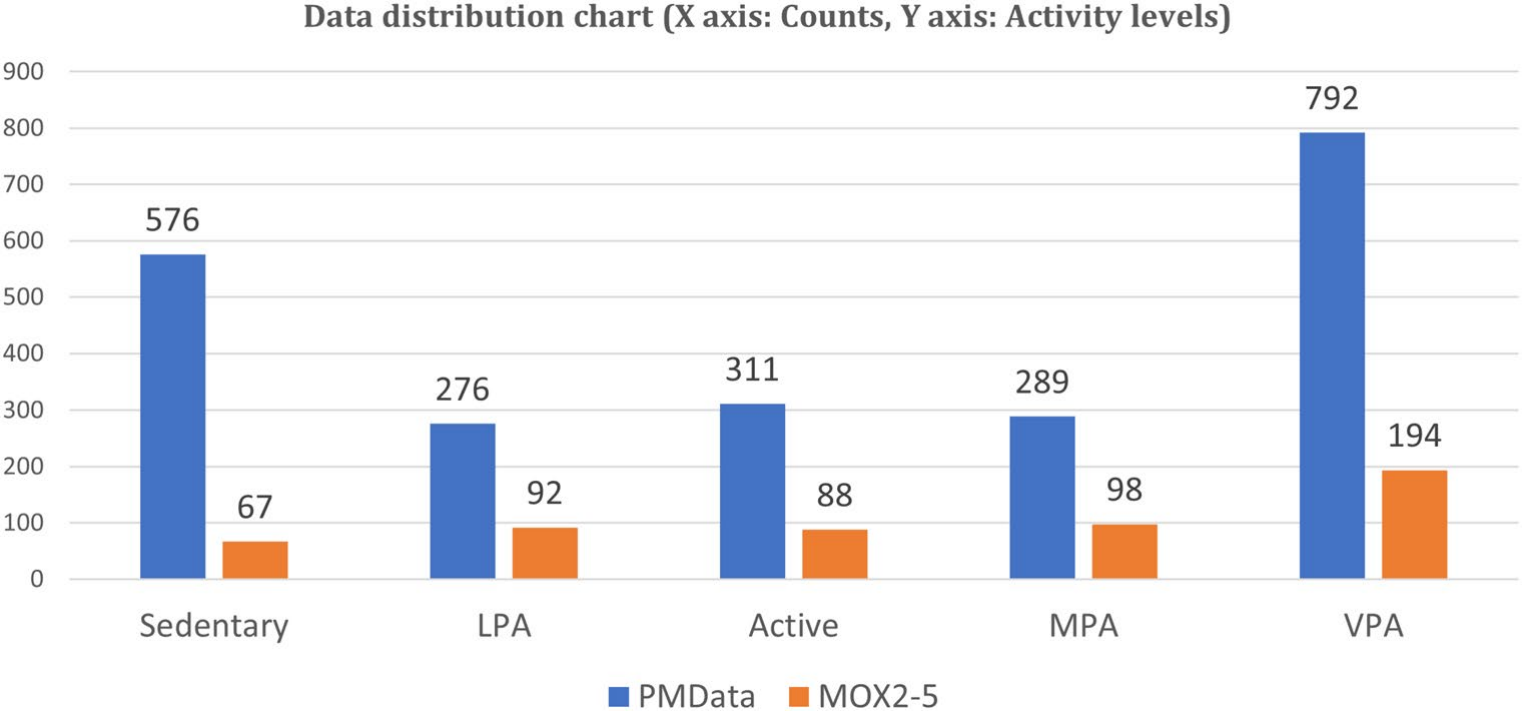
The comparison of the distribution of classes for the public PMData and the private MOX2-5 datasets.

### Data labelling for classification

The activity level characteristics represent the following five categories—Sedentary (0), Low Physical Active (1), Active (2), Moderately Physical Active (3), and High Physical Active (4). Activity level feature class creation rules are defined in Table 4, where we derive feature classes based on sedentary lifestyle, LPA, MPA, and VPA by adult activity reference^5,8,48,49^. Characteristics such as age, sex, and weight were not the subject of this study. The class distributions of the two datasets are shown in Fig. 3.

### Deep learning time-series classifier

The architecture of the time-series classifier we developed is inspired by standard, well-known MLP architectures based on the fully connected neural network (FCNN) style. Since our dataset is small, we employed a decent number of neurons in each layer based on common heuristics (e.g., validation loss, hidden units are a fraction of the input). The entire sequential structure of the model we developed consists of six fully connected dense layers, an input layer $\in$ $R^{32}$, followed by a hidden layer $\in$ $R^{32}$, then, three hidden layers $\in$ $R^{16}$ followed by an output layer $\in$ $R^{5}$. The input dimension of the input layer is five. Due to the limited number of functions and data, regularization and dropout layers are not used. We checked; however, L1 and L2 regularizers could not help much to improve the model performance.

For the first five layers, we chose the rectified linear unit (ReLU) activation function over other linear and nonlinear functions because ReLU does not have the zero gradient problem and generally leads to faster convergence^50^. We used the SoftMax activation function in the last layer to classify the data according to the probability distribution. The expression for the ReLU is

5 $$\begin{aligned} Relu(z) = max(0, z). \end{aligned}$$

We used the categorical$\_$crossentropy loss function in model compilation because we one-hot encoded the predictor class variables. Also, we used the ADAM optimizer because it is computationally efficient and consumes less memory. The ADAM configuration parameters are $\alpha$ (the learning rate), $\beta _1$ (the exponential decay rate of the first moment guess), $\beta _2$ (the exponential decay rate of the second moment guess), and $\epsilon$ (very Small numbers to prevent division by zero). In Keras, the default ADAM configuration is $\alpha$=0.001, $\beta _1$=0.9, $\beta _2$=0.999, $\epsilon$=1e-08 and Decay=0.0, and this experiment also uses the same configuration. We used validation split = 0.05, verbose = 0, and the callback of ReduceLROnPlateau to reduce the learning rate and improve the model’s performance. We recorded loss histories to compare training and test losses over multiple epochs.

### Deep learning univariate time-series forecasting

CNNs are primarily designed and developed to process two-dimensional (2D) image data. However, CNNs can automatically extract and learn features from one-dimensional sequence data, such as patterns in univariate time-series data. The traditional, well-known CNN architecture inspired the univariate predictive model we developed. Since our dataset is small, we kept a reasonable number of neurons in each layer based on common heuristics (e.g., validation loss, hidden units are a fraction of the input). The model’s overall structure consists of the following five layers-two CNN1D layers, one MaxPooling1D layer, one flattening layer, and one dense seed layer. A Conv1D layer consists of 3D input and output tensors of shape (Batch, Steps, Channel) and (Batch, $new_{steps}$, Filter), respectively. The output shape changes depending on padding or stride selection. The batch dimension is the number of samples in the dataset, which is called “None” because it is not fixed. We performed linear convolution operation using Keras Conv1D plane with input parameters filter $kernel_{size}$ and padding.

Due to the limited number of functions and data, the dropout layer is not used. MaxPooling1D blocks sample input data, parameters, and computed convolutions needed to control overfitting. The flattened layer takes compressed input from a MaxPooling1D block and converts the data into 1D linear vectors for input to the following dense layer. We used the standard MaxPooling1D parameter defined in the Keras library^51^. We kept the kernel size of the CNN1D layer as 3. We used a sequential model with two CNN1D layers, a MaxPooling1D layer, and a flattened and dense output layer with an output size of 1. We chose the ReLU activation function for the first two CNN1D slices to avoid vanishing gradients and achieve faster convergence.

We used public PMData and private MOX2-5 datasets for model training, testing, and cross-validation. Before training, we processed our active dataset with MinMaxScaler ($\mu$ = 0 and $\sigma$ = 1) with features ranging between 0 and 1. We then calculated a timestep value as the difference between the training set’s length and the training data’s size. The time steps are valued as n$\_$steps, n$\_$features = 1. The input form of the initial CNN layer consists of the following two input parameters: n$\_$steps and n$\_$features.

We used the mean squared error (MSE) loss function to compile our CNN1D model because we performed one-hot encoding on the predictor class variables. Also, we used the ADAM optimization function because it is computationally efficient and consumes less memory. Adam optimization is a stochastic gradient descent method based on adaptive first and second-moment estimation. We used the standard ADAM configuration parameters available in Keras. We used validation split = 0.05, verbose = 0, and the callback of ReduceLROnPlateau to reduce the learning rate ($\alpha$) and improve the model’s performance.

We compared our developed CNN1D model with other baseline predictive models such as autoregressive (AR), LSTM, and GRU. We evaluated each model for 200 epochs with a stack size of 50. We used 100 neurons for the LSTM and GRU base models, the ADAM optimizer, and the MSE loss function for model compilation. The AR time series base model was improvised with residual error minimization (REM) to verify how our model solves the traditional REM problem in time series step data. We created a lag value of 50 for the PMData dataset and 14 for the MOX2-5 dataset. We consider two datasets with AR window lengths 5.

### Interval prediction over point prediction

In predictive inference, a prediction interval estimates a gap in which future observations will have some probability of falling, assuming what has already been observed^52,53^. Prediction intervals are often used in prediction analysis. In this study, we used the concept of step forecasting. The prediction interval, which gives the gap to maintain a specific probability value, can be written as

6 $$\begin{aligned} {\widehat{Y}}_{T+h} \pm c\sigma _{h}. \end{aligned}$$

*c* changes with coverage probability. In 1-step interval prediction, *c* is 1.28 (80$\%$ prediction interval where forecast error values are normally distributed). $\sigma _{h}$ estimates the residual standard deviation in the h-step forecast distribution ($h>0$). Residual standard deviation (RSD) statistically describes the difference between the standard deviation of observed values and the standard deviations of estimated values. We used a well-accepted Naïve forecast method to statistically derive “$\sigma _{h}$” under the assumption of uncorrelated residuals.

### Ontology processing

In Fig. 2, the TDB database acts as a KB. All the messages as described in Table 6 are stored in the KB. The RG module in Fig. 2 is used to access these messages during tailored recommendation generation based on SPARQL query execution, followed by implementing the rules in Table 5. The rules are also stored in the KB. The asserted and inferred knowledge obtained from the reasoning method helped determine the most suitable recommendation message. Ontology models are associated with a document manager, OntDocumentManager to assist in processing ontology documents. All classes that represent the value of the ontology in the ontology API have OntResource as a general superclass. We have implemented the RDF interface provided by Apache Jena to persist the designed and developed OntoeCoach ontology and its instances in the TDB and load them back for further processing. Jena Fuseki is tightly integrated with TDB to provide a robust transactional persistent storage layer. The reasoning time of the OntoeCoach ontology is measured against the following reasoners available in the Protégé: HermiT, KAON2, Pellet, RacerPro, and Fact++.

### Performance evaluation

We utilized multiple state-of-the-art metrics to evaluate and compare the performance of the classifier, forecasting, and OntoeCoach models.

#### Classification

The performance of DL-based multi-class classification models was evaluated against discrimination analysis. Multiple metrics such as classification report, confusion matrix, precision, recall, specificity, accuracy score, and F1 score were estimated^2^. A confusion matrix is a 2-D table (*actual* versus *predicted*) and both dimensions have four options, namely, *true positives (TP)*, *false positives (FP)*, *true negatives (TN)*, and *false negatives (FN)*. *TP* is an outcome where the model estimates the positive class accurately; *TN* is an outcome in which the model correctly predicts the negative class; *FP* is an outcome where the model estimates the positive class inaccurately; and *FN* is an outcome in which the model predicts the negative class incorrectly. The corresponding equations are −

7 $$\begin{aligned}{} & {} Precision = \frac{TP}{TP + FP} \end{aligned}$$

8 $$\begin{aligned}{} & {} Recall = Sensistivity = \frac{TP}{TP + FN} \end{aligned}$$

9 $$\begin{aligned}{} & {} Accuracy = \frac{TP+TN}{TP+TN+FP+FN} \end{aligned}$$

10 $$\begin{aligned}{} & {} F1\text {-}score = \frac{2 \times Recall \times Precision}{Recall+Precision} \end{aligned}$$

A higher value from the above expressions represents a better performance of a model, and this applies to all performance metrics. On the other hand, *bias* is an error due to erroneous assumptions in the learning algorithm, and *variance* is an error from sensitivity to small fluctuations in the training set. While high bias leads to under-fitting, high variance results in overfitting. *Accuracy* and *F1-scores* can be misleading because they do not fully account for the sizes of the four categories of the confusion matrix in the final score calculation. In comparison, the *MCC* is more informative than the *F1-score* and *Accuracy* because it considers the balanced ratios of the four confusion matrix categories (i.e., *TP, TN, FP*, and *FN*). The *F1-score* depends on which class is defined as a positive class. However, *MCC* does not depend on which class is the positive class, and it has an advantage over the *F1-score* as it avoids incorrectly defining the positive class^54^. The *MCC* is expressed as follows^38^.

11 $$\begin{aligned} \small MCC = \frac{TP*TN - FP*FN}{\sqrt{(TP+FP)(TP+FN)(TN+FP)(TN+FN)}} \end{aligned}$$

#### Forecasting

The performance of each time-series forecasting model was evaluated with root mean squared error (RMSE). MSE informs how close the regression line is to a set of points. It calculates “errors” from the points to the regression line and squares them to eliminate negative signs. The squared root of MSE gives more weight to a significant difference with no bias^45^. The RMSE can be expressed as ($y_{i}$ represents the predicted value and $x_{i}$ represents the expected value)

12 $$\begin{aligned} RMSE = \sqrt{(\frac{1}{n})\sum _{i=1}^{n}(y_{i} - x_{i})^{2}}. \end{aligned}$$

Additionally, we have used other metrics such as Forecast Bias (FB), RSD, and model execution time in seconds (s). FB can be positive or negative. A nonzero mean forecast error value indicates the tendency of the model to overpredict (negative error) or underpredict (positive error). Therefore, the average forecast error is also called FB. If Forecast Error = 0, the forecast has no errors or perfect predictive power. Overpredict if forecast variance < 0, the model is unbiased if forecast variance $\approx$ 0^45^.

#### Statistical

We developed new four statistical metrics beyond the existing ones. (1) activity pattern vector (APV)—a weekly activity pattern vector of length 7 that contains an activity level score for a given week. Thus, it can also be termed as an activity level vector (ALV), (2) similarity score (SC)—a weekly similarity score is a difference between the summation of the weekly activity pattern vector and weekly goal vector. If SC $\ge$ 0, then it signifies that the participant has achieved a weekly goal, (3) weighted mean ($\mu _{S}$)—standard mean calculation with weighted mean calculation to determine personal activity intensity on a weekly basis and thereby use the information in activity recommendations (e.g., based on the progress, the activity on Week-2 will likely match the action performed; however, your activity was very good on Week-3). We calculated a weighted mean on an individual weekly activity dataset to calculate weekly activity progression with a defined non-negative weight point set: $\{0, 2, 4, 6, 8\}$ that represents sedentary, low active, active, medium active, high active, (4) standard deviation ($\sigma$)—weighted mean values to calculate deviations in weekly activity intensities.

We evaluate these statistical metrics using the following steps. *Step 1*—load individual activity datasets for the last few weeks, *Step 2*—calculate the weekly mean of the following activity features F: Sedentary time, LPA, MPA, VPA, Steps, *Step 3*—calculate weekly activity level score based on the activity level classification results, APV, *Step 4*—SC = $\Sigma$ APV ($W_i$) - $\Sigma$ GoalScore ($W_i$), where $W_i$ signifies a week, *Step 5*—calculate performance score against APV with the following rule: Performance Score (S) = $\Sigma$ activity level on day-n * activity weight point ($point_i$), *Step 6*—$\mu _{S}$ = Calculate the mean of S on weekly basis (= S/7), *Step 7*—predict or calculate activity intensity of the corresponding week based on $\mu$ score and prepare a weightedMeanList, and *Step 8*—calculate deviation in between weekly activities and prepare a deviationList.

#### Ontology

Our proposed ontology model was evaluated against the following two metrics reasoning time, and query execution time. Protégé provides a list of reasoners, such as HermiT, Fact++, Pellet, KAON2, and RacerPro, to check the logical and structural consistencies. We compared mean reasoning time and selected the best reasoner for our ontology. Besides, we captured the SPARQL query execution time in Protégé. We loaded the ontology file in “TTL” format into the Jena Fuseki server for cross-verification in SPARQL query execution time. We used the Apache Jena Framework to query each ontology class, predicate, subject, and object.

#### Ethical approval and consent to participate

In this project, for handling personal health and wellness data, we received approval from the Norwegian Centre for Research Data (NSD) (797208) and we obtained ethical approval from the Regional Committees for Medical and Health Research Ethics (REK) (53224). For this study, participation has been voluntary, and informed or signed consent has been obtained from all the participants. Moreover, we have not disclosed any identifiable data of the participants using numbers, text, or figures.

## Results

We performed the complete experiment on PMData and MOX2-5 datasets for verification. The volume of the PMData dataset was more than the MOX2-5 datasets.

### Correlation analysis and feature ranking

**Figure 4:**
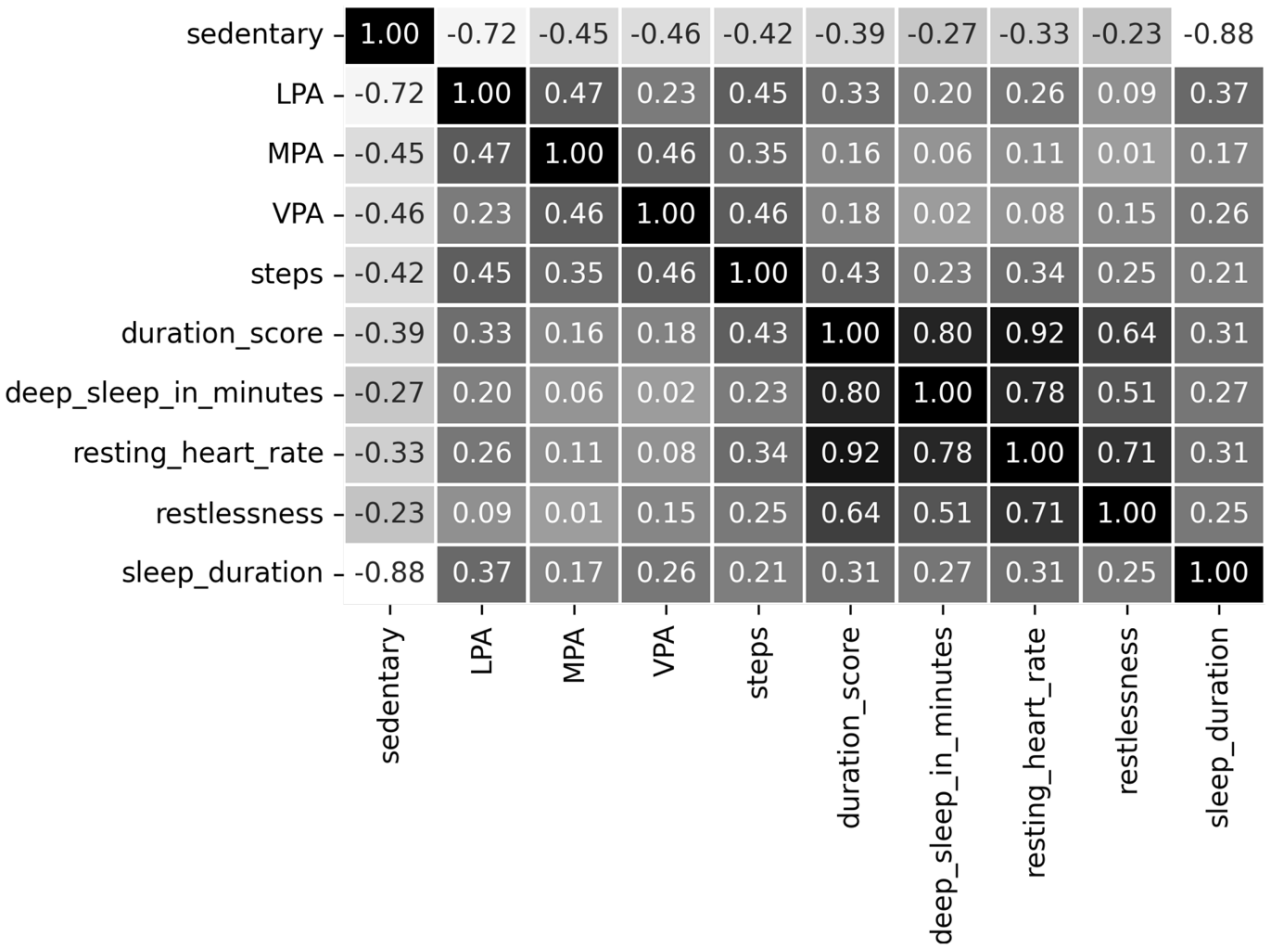
The feature correlation in the PMData datasets.

**Figure 5:**
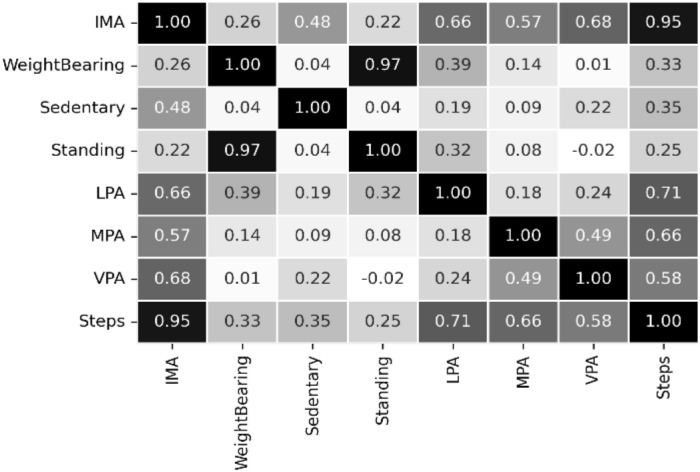
The feature correlation in the MOX2-5 datasets.

The correlation matrix of the features selected from the PMData and MOX2-5 datasets are depicted in Figs. 4 and 5, respectively. The resultant |*r*| value helps to understand the strong association between the features, followed by preparing the final feature set to run the entire experiment. We found that the duration$\_$score, resting$\_$heart$\_$rate, deep$\_$sleep$\_$in$\_$minutes, and sleep$\_$duration features produced a very high correlation in the PMData dataset. Whereas IMA, standing, and WeightBearing features produced a very high correlation in the MOX2-5 dataset.

Moreover, we prepared the final feature set for daily activity level classification, with the most relevant features, such as Steps, sedentary, LPA, VPA, and MPA, based on the adopted feature analysis methods, such as SelectKBest, PCA, and ExtraTreeClassifier. The selected features are presented in Table 10 for both datasets based on their ranks. Table 10 reveals that in both the datasets the “Step” feature has achieved the highest rank against the used methods.

**Table 10:** The feature ranking in datasets against different methods.

Method	Datasets and rankings
SelectKBest	PMData: steps, sedentary, LPA, VPA, MPA and MOX2-5: steps, sedentary, LPA, VPA, MPA
PCA	PMData: steps, VPA, MPA, LPA, sedentary and MOX2-5: steps, VPA, MPA, LPA, sedentary
ExtraTreesClassifier	PMData: steps, VPA, sedentary, LPA, MPA and MOX2-5: steps, LPA, MPA, VPA, sedentary

**Table 11:** Classification results on PMData datasets.

Models	Precision (%)	Recall (%)	F1-score (%)	Accuracy (%)	MCC (%)
Our MLP model	97.0	97.0	97.0	97.0	94.0
Rocket	51.0	56.0	52.0	56.0	54.0
MiniRocket	66.0	52.0	58.2	58.2	54.2
MiniRocketVoting	45.0	52.0	48.5	49.0	46.0

**Table 12:** Classification results on MOX2-5 datasets.

Models	Precision (%)	Recall (%)	F1-score (%)	Accuracy (%)	MCC (%)
Our MLP model	74.0	71.0	72.5	71.0	69.0
Rocket	56.0	42.0	48.0	48.0	45.0
MiniRocket	58.0	45.0	50.2	51.0	49.0
MiniRocketVoting	39.0	44.0	41.3	42.0	41.0

### Classification performance

The performance of our developed time-series classifier and other state-of-the-art time-series classifiers, such as Rocket, MiniRocket, and MiniRocketVoting, was evaluated for both PMData (see Table 11 and MOX2-5 (see Table 12) datasets. The proposed MLP classifier model has outperformed other baseline state-of-the-art classifiers for both PMData and MOX2-5 datasets with an accuracy score of 97.0$\%$ (precision=97.0$\%$, recall=97.0$\%$, F1-score=97.0$\%$), and 74$\%$ (precision=71.0$\%$, recall=72.5$\%$, F1-score=71.0$\%$), respectively. The MLP model has produced the best performance on selected features in the low-volume activity datasets.

**Figure 6:**
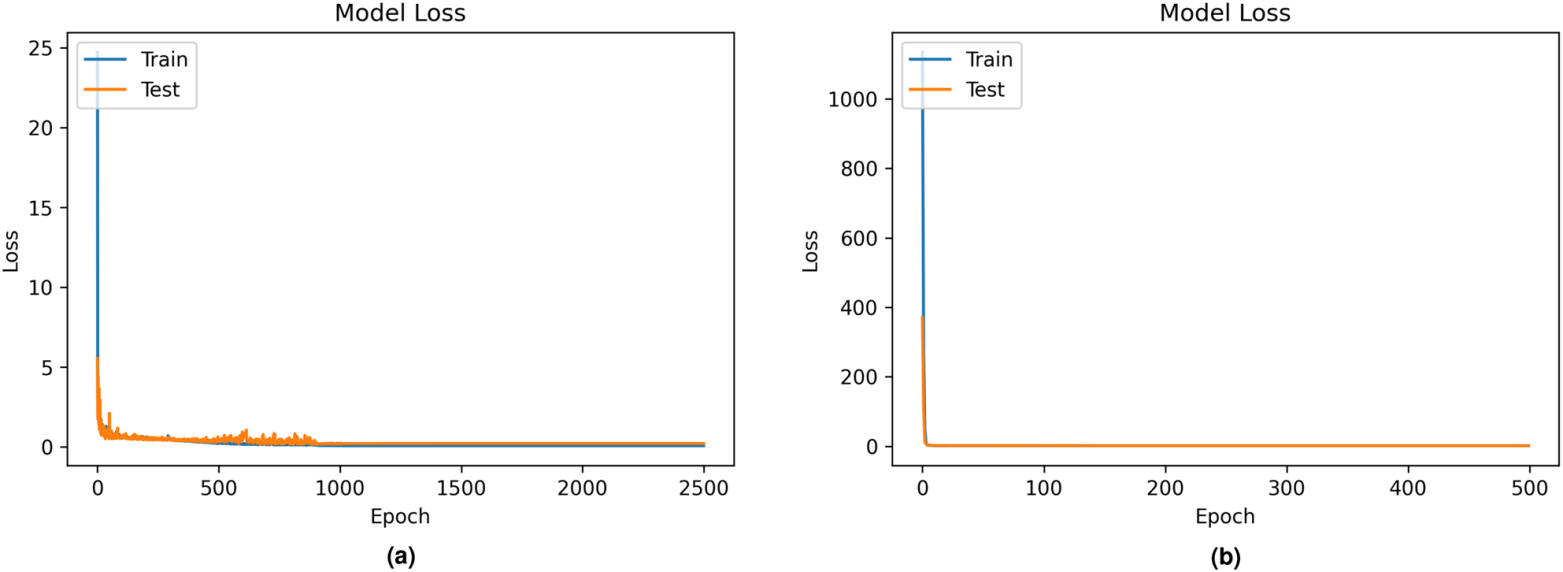
Model Loss of our proposed classifier in PMData (**a**) and MOX2-5 (**b**) datasets.

**Figure 7:**
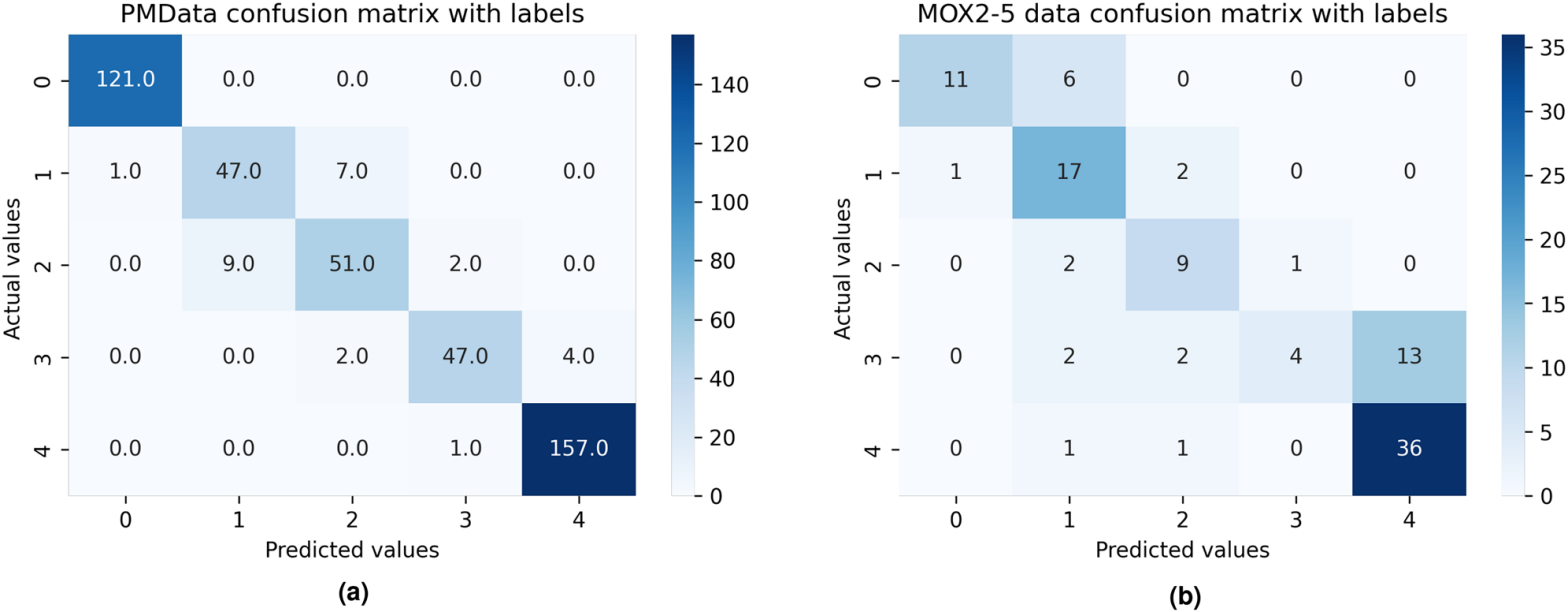
The confusion matrix in the classification of PMData (**a**) and MOX2-5 (**b**) datasets with a weighted average precision, recall, and accuracy score.

**Table 13:** Mean step forecasting results on PMData datasets.

Models	RMSE	| FB |	RSD	ET (s)
Our CNN1D	1520.9	222.54	1534.0	88.0
AR with REM	5936.5	223.4	1475.6	144.0
Vanilla LSTM	4537.3	234.0	4574.7	149.2
Stacked LSTM	4541.7	244.0	4580.4	232.6
Bidirectional LSTM	4369.7	369.0	4411.0	211.8
Vanilla GRU	4488.3	223.5	4526.6	146.8
Stacked GRU	4518.6	125.0	4515.0	234.2
Bidirectional GRU	4367.4	224.6	4434.3	219.3

**Table 14:** Mean step forecasting results on MOX2-5 datasets.

Models	RMSE	| FB |	RSD	ET (s)
Our CNN1D	1742.7	246.3	1796.3	88.0
AR with REM	3753.1	150.0	3956.4	143.0
Vanilla LSTM	3831.5	128.4	3951.0	157.3
Stacked LSTM	3788.7	111.0	3907.2	199.3
Bidirectional LSTM	3687.9	138.0	3801.7	192.0
Vanilla GRU	3930.9	104.8	4052.9	152.0
Stacked GRU	3877.1	185.3	4007.1	205.5
Bidirectional GRU	3703.9	117.5	3819.4	209.3

**Table 15:** Statistical analysis on last 4 weeks’ STATISTICAL ANALYSIS ON LAST FOUR WEEKs’ ACTIVITY DATA FOR P-1 IN MOX2-5 DATASETS. *LPA* Low physical activity, *MPA* Medium physical activity, *VPA* Vigorous physical activity, *APV* Activity pattern vector, *GS* Goal score, *SC* Similarity score, *AP* Activity point, *S* Activity performance score, *SD* Standard deviation.

Metrics	Week-1	Week-2	Week-3	Week-4
Mean sedentary time (s)	2146.0	81,838.0	91,305.0	940.0
Mean LPA time (s)	5935.0	3799.0	2551.0	3240.0
Mean MPA time (s)	1239.0	1008.0	316.0	682.0
Mean VPA time (s)	55.0	164.0	0.0	383.0
Mean steps	11,706	8861	4649	7256
APV	[3, 3, 3, 4, 4, 2, 4]	[4, 4, 4, 2, 0, 0, 0]	[0, 0, 0, 0, 0, 1, 0]	[1, 1, 3, 2, 1, 1, 1]
GS	[3, 3, 3, 3, 3, 3, 3]	[3, 3, 3, 3, 3, 3, 3]	[3, 3, 3, 3, 3, 3, 3]	[3, 3, 3, 3, 3, 3, 3]
SC	+ 2	− 7	− 20	− 11
$\Sigma$AP	158	104	2	36
Mean S	22.5	14.9	0.3	5.1
Weekly (|*SD*|)	0.0 (Error: ± 0.0)	3.8 (Error: ± 2.7)	9.2 (Error: ± 5.3)	8.6 (Error: ± 4.3)

**Table 16:** Step and interval prediction for Week-X for P-1 in MOX2-5 datasets.

Week-x	Predicted step points (SP)	80$\%$ interval step prediction with c = 1.28, $\sigma _h$ = 1271.0
Day-1	3520.0	[1893, 5147]
Day-2	5171.0	[3544, 6798]
Day-3	4855.0	[3228, 6482]
Day-4	4979.0	[3353, 6605]
Day-5	5071.0	[3445, 6697]
Day-6	4508.0	[2882, 6134]
Day-7	3928.0	[2302, 5554]

**Table 17:** Performance comparison of different ontology reasoners available in Protege.

Reasoner(s)	Average reasoning time (s)
HermiT	1.0–2.0 s
Pellet	2.0–4.0 s
Fact++	3.0–4.0 s
RacerPro	2.0–3.0 s
KAON2	3.0–4.0 s

We compute the model loss for both datasets. The loss value indicates how well the model performed after each optimization iteration. It is a value representing the sum of the errors in our developed MLP classifier model. Loss measures how well (or poorly) our model performs. The “Model Loss” with categorical entropy to compare training and test sets over epochs for both the datasets have been depicted in Fig. 6 together with the confusion matrices in Fig. 7 to describe the weighted average precision, recall, and accuracy score for both datasets against our developed MLP classifier.

Results in Figs. 6 and  7 show that MLP model loss in training and testing data converges for both datasets without showing any abruption or divergence. The confusion matrices provide insight not only into the incorrect classifications of developed MLP classifiers but also into the types of mistakes made. According to the confusion matrices, the performance of the MLP classifier increases with more training data. Therefore, misclassification rates are less in PMData datasets as compared to MOX2-5 datasets. Similar precision and recall scores signify that FP = FN and their similarity with accuracy tells that our developed MLP model is balanced. However, this may vary from cases and datasets. DL models improve their learning with an increased volume of data. The evidence has been captured in Tables 11 and 12. The proposed MLP classifier has outclassed its nearest best-performing MiniRocket classifier with $\approx$ 46$\%$ and 27.5$\%$ accuracy improvement for PMData and MOX2-5 datasets, respectively.

### Prediction outcomes

The mean performance analysis against forecasting matrices between our CNN1D-based univariate “Step” forecasting model and other existing DL forecasting models has been compared in Tables 13 and 14 for both datasets. Our developed CNN1D model reduces the RMSE error, improves forecast bias, and balances residual standard deviation for both datasets. Forecasting results in both tables show that our developed CNN1D has outperformed other baseline time-series forecasting models against state-of-the-art evaluation matrices. Its close competitors are bidirectional LSTM and GRU models. We found that the CNN, LSTM, and GRU effectively manage residual errors, and produce better results than AR with the REM technique.

### Statistical analysis and interval prediction

Based on the proposed weighted mean calculation method, we showed the weekly activity score (S), similarity score (SC), and standard deviation (SD) calculation for participant-1 or P-1 from the MOX2-5 datasets in Table 15. For example, we considered the activity data of P-1 for the last 4 weeks. We can use the same method for other participant data. The mean sedentary, LPA, MPA, and LPA times are measured in seconds. SC signifies that P-1 has failed to achieve weekly goals for the last three consecutive weeks and therefore needs proper recommendation planning to stay motivated in the following weeks. The S and SD values tell that the activity performance has significantly dropped after Week-1.

Moreover, we used our CNN1D model for the next 7 days’ step forecast for P-1 based on its temporal step data analysis. We calculated the RSD value $\approx$ 1271.0 for the step data of P-1. Using the Naïve-based interval prediction method, we showed a direction to calculate the 1-step interval prediction of activity steps on top of the point prediction (see Table 16). The mean predicted steps for the following week (Week-X) produced a value of 4576.0 ($\approx$ (3520.0 + 5171.0 + 4855.0 + 4979.0 + 5071.0 + 4508.0 + 3928.0)/7) which tells that the upcoming week (or Week-X) can be a match with Week-3. Therefore, the daily activity performance must be improvised.

### Query execution and recommendation generation

We generated personalized activity recommendations during ontology validation based on semantic rules to improve individual physical activity levels to achieve activity goals. We executed semantic rules and used the Jena ARQ engine to run associated SPARQL queries on the used dataset. Query results have been combined to create tailored recommendations to meet the eCoaching requirements. For instance, in Week-3, participant P-1 failed to achieve WHO’s generic activity goal to stay active. Therefore, based on the semantic rule, he received recommendation messages A-19 and A-17. Based on the step forecast results with our developed CNN1D model, P-1 received recommendation message A-13 for the following week. On Week-3, the set of daily classified activity levels or APV is [0, 0, 0, 0, 0, 1, 0]. Therefore, for activity level 0, P-1 received A-1, A-7, A-8, A-10, and A-15, and for activity level 1, P-1 received A-2, A-7, A-8, A-10, and A-15.

We utilized the OWL$\_$MEM$\_$MICRO$\_$RULE$\_$INF specification (OWL-full) to investigate the ontology structure in Jena in the TTL format and approximated the reading time to 1.0–1.5 s. Moreover, we used In-memory storage, optimized rule-based reasoner OWL rules, and the Jena framework to query the ontology class, ontology, predicate, subject, and object of each sentence in < 1.0 s, < 2.0 s, and < 2.0 s, respectively. The reasoning time of the OntoeCoach ontology has been captured in Table 17. The HermiT reasoner performed the best without any inconsistencies.

**Figure 8:**
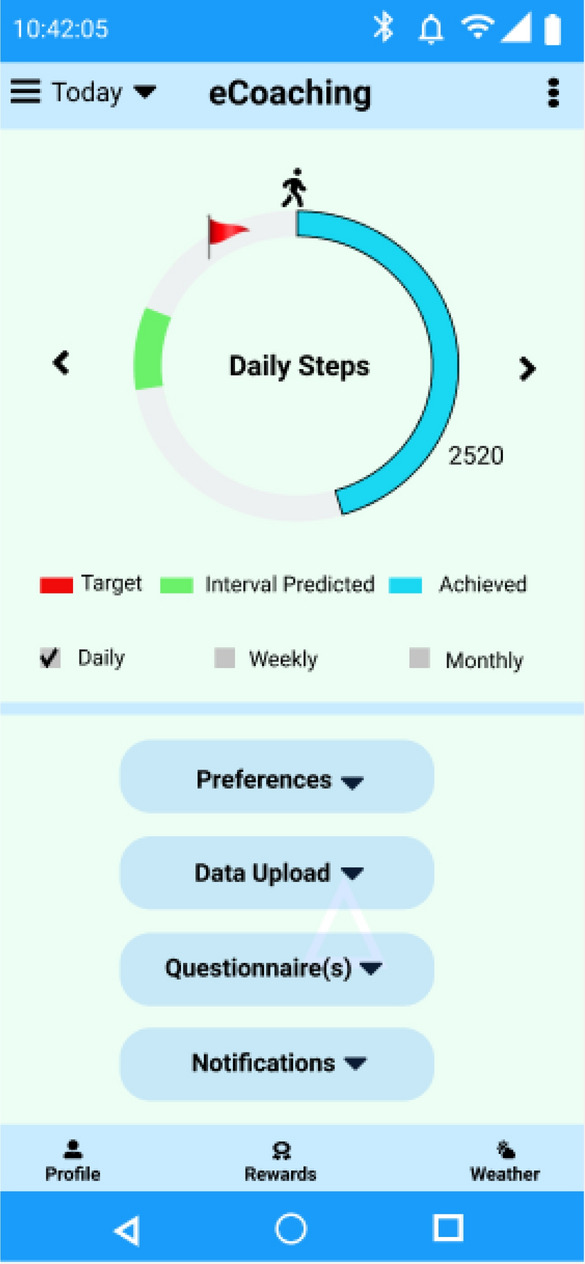
Visualization of daily step count, target step count, and predicted interval.

## Discussion

This work presents a novel deep learning and ontology-based personalized recommendation modeling and includes comprehensive and multiple comparison levels to appreciate the proposed approaches’ performance better. From the classification and forecasting results on both datasets, we found that DL models for time-series prediction and classifications can be effectively designed and developed. Further, we integrated these models in the OntoeCoach model for hybrid personalized recommendation generation.

According to the evidence in Tables 11 and 12, an increased volume of MOX2-5 datasets could improve our model performance in this multi-class classification problem. In both datasets, model loss for training and testing converges. Due to the higher volume in PMData as compared to MOX2-5 datasets, our MLP classifier took more epochs for convergence. We compared the result of our proposed MLP classifier with traditional ML classifiers, such as SVM with linear and non-linear kernels, Decision Tree, KNN, Naïve Bayes, LDA, and our model outperformed these ML classifiers on PMData datasets. We planned to perform a similar comparison on MOX2-5 datasets in our future study with increased data.

Across both datasets, CNN1D outperformed other forecast models and produced high-speed output. We tried to increase the efficiency of the CNN1D model with more hidden layers, neurons, variations in filters, and dropout layers; however, we could not succeed. A limited volume of datasets can be a strong reason behind this. We also noticed that CNN, LSTM, and GRU models have different hyperparameters in terms of filter dimension, the number of filters, and hidden state dimension, and they internally work differently. CNN1D generally manipulates the spatial correlation in data and performs well when capturing the neighborhood information in data.

Future step prediction for individuals combined with the estimated S-value for the previous weeks can be a good direction for generating tailored recommendations. Similar studies are missing in the literature. Figure 8 shows a visual approach to present the interval step prediction in the ActiCoach smartphone application to motivate individuals to personal activity monitoring to reach their activity goals.

Average execution times for SPARQL queries were recorded between 0.1 and 0.4 s. The semantic rules described in Table 5 represent the logic behind generating personalized recommendation messages. Rule-Based binary reasoning (if $\rightarrow$ 1 else $\rightarrow$ 0) helps to explain the formation of personal activity recommendation messages. A complete data-driven approach to personalized recommendation generation in healthcare is still critical due to false-positive scenarios. Therefore, prediction modeling followed by an annotated ruleset can add more value to personalized health recommendations. For solving the generic cold-start problem in our personalized recommendation generation, we recorded data for an initial two weeks to identify the activity patterns in an individual before starting DL-based data processing and followed by a recommendation generation.

Our modular eCoach system design can integrate other ML and DL classifiers, predictors, and statistical methods (e.g., daily activity frequency, graded activity frequency, regular activity frequency, and distribution of daily activity patterns). In that case, we only need to update respective models and techniques. The concept of ontology supports new branching to integrate new ideas or pruning if some ideas are unnecessary. The KB and RMT can grow or shrink on demand based on future studies’ efficacy evaluation. Furthermore, this type of design approach can support similar activity sensors (e.g., Actigraph).

This study proves an integrated concept for hybrid personalized recommendation generation in activity eCoaching, combining time-series classification and forecasting results with semantic ontology to generate rule-based personalized recommendations. However, a longitudinal study on a group of controlled trials could evaluate its practical efficacy. More state-of-the-art time-series models (classification and forecasting) for performance comparison, stability analysis, and more activity attribute support with the growing activity data can be included. The recommendation generation performance could improve by using density-based spatial clustering, sessions, criteria, similarity score, reward maximization, fuzzy logic, entropy, and community-based heuristic approaches. In the current approach, a person can receive multiple recommendation messages. Thus, the scope of the solution can be increased with meta-heuristic methods to select an optimal set of recommendations from a feasible recommendation set and make the selection dynamic with personal behavioral patterns.

Collaborative filtering is a well-established recommendation method to generate recommendations to filter out items based on the user similarity score. It defines an optimal search space that includes users with the closest preference score. The similarity score helps to create profile rankings. Our model-based exercise recommendations are filtered based on personal preferences and short- and/or long-term goal achievement. The tree structure of the semantic ontology explains the binary logic or rules behind specific recommendation generation. The process is highly individualized; thus, the notion of group similarity is not included in recommendation generation. In the future, we will extend this research to group-based meta-heuristics by incorporating ideas from collaborative filtering.

The proposed Activity eCoach system demonstrates its significance in real life by offering personalized guidance, support, and motivation to individuals aiming to enhance their physical health and overall well-being. Our physical activity eCoaching system could offer multiple benefits and use cases in the real world, as demonstrated by real-life examples (a–j). These could directly contribute to the sustainable development goal of the nation e.g., the United Nations’ Sustainable Development Goal (SDG) 3^55^. (a) *Personalized Approach*: Our activity eCoaching offers a personalized approach to fitness and wellness. It takes into account individuals’ unique characteristics, goals, preferences, and constraints, allowing for tailored recommendations and strategies that align with their specific needs. This personalized approach enhances engagement and increases the likelihood of successful behavior change. (b) *Accessibility and Convenience*: Our activity eCoaching provides accessibility and convenience to individuals. With the use of mobile applications, online platforms, and wearable devices, individuals can access coaching support and resources anytime, anywhere. This flexibility eliminates geographical barriers and time constraints, making it easier for people to engage in fitness activities and receive guidance, regardless of their location or schedule. (c) *Continuous Support and Accountability*: Our activity eCoaching provides continuous support and accountability. Coaches can monitor individuals’ activity progress, track their activities, and provide timely feedback and encouragement. This ongoing support helps individuals stay motivated, overcome obstacles, and maintain consistency in their fitness journey. (d) *Goal Setting and Progress Tracking*: Our activity eCoaching facilitates goal setting and progress tracking. Activity eCoache system works with individuals to set realistic and achievable goals, breaking them down into manageable steps. Regular tracking of progress allows individuals to visualize their achievements, identify areas for improvement, and make necessary adjustments to their routines. (**e**) *Education and Guidance*: Our activity eCoach system can provide evidence-based information, answer questions, and address concerns, empowering individuals to make informed decisions about their health and well-being. (f) *Behavior Change Support*: Our activity eCoaching focuses on behavior change strategies and techniques. eCoaches help individuals develop new habits, overcome barriers, and adopt healthier lifestyles. They provide guidance on setting realistic expectations, managing setbacks, and sustaining long-term behavior change. (g) *Motivation and Engagement*: Our activity eCoaching enhances motivation and engagement. Through personalized feedback, progress updates, goal achievements, and interactive features, individuals are motivated to stay active and engaged in their fitness routines. Recommendation and rewarding features further enhance motivation and create a sense of community. (h) *Health Monitoring and Risk Management*: Our activity eCoaching can incorporate health monitoring features to track vital health signs, heart rate, sleep patterns, and other relevant health indicators. This may allow to identify potential health risks, provide early intervention, and promote overall well-being. (i) *Integration with Other Healthcare Services*: Our activity eCoaching can be integrated with other healthcare services, such as telemedicine or electronic health records, to ensure a comprehensive approach to individuals’ health management. eCoaches may collaborate with healthcare providers, share relevant data, and align coaching strategies with medical recommendations. (j) *Long-Term Sustainability*: Our activity eCoaching aims to promote long-term behavior change and sustainability. Providing ongoing support, education, and personalized strategies, eCoaches help individuals develop healthy habits that can be sustained beyond a specific program or intervention.

## Conclusion

To improve an individual’s physical activity levels through wearable activity sensors and digital activity trackers, eCoach capabilities may be encouraging. Through continuous monitoring and personalized recommendation generation, eCoach can motivate participants to achieve their physical activity goals to maintain a healthy lifestyle. This work proposes a new theoretical concept for generating personalized activity recommendations in eCoaching using a hybrid approach. The idea of univariate time series forecasting exists; its application to the ontology of activity eCoaching and interval forecasting is novel. This study reveals a method for examining and using projection, classification, statistical, and recommendation models with semantic rule bases to design and develop a prototype eCoach system to generate interpretable and personalized campaign recommendations to manage campaign goals.

## Data Availability

The corresponding author AC can be contacted for the datasets and codebase.

## Abbreviations

eCoach: Electronic coaching
ICTs: Information and communication technologies
ML: Machine learning
DL: Deep learning
SWRL: Semantic web rule language
SPARQL: SPARQL query protocol and RDF query language
PoC: Proof-of-concept
OWL: Web ontology language
RDF: Resource description framework
RDFS: RDF schema
KB: Knowledge base
AI: Artificial intelligence
LTG: Long-term goals
STG: Short-term goals
LPA: Low physical activity
MPA: Medium physical activity
VPA: Vigorous physical activity
SVM: Support vector machine
NB: Naive Bayes
KNN: K-nearest neighbour
DT: Decision tree
PCA: Principal component analysis
LDA: Linear discriminator analysis
MLP: Multi-layer perceptron
CNN: Convolution neural network
RNN: Recurrent neural network
LSTM: Long short-term memory
GRU: Gated recurrent unit
ReLU: Rectified linear unit
RMSE: Root mean squared error
AR: Auto regression
TDB: Touple database
NSD: Norwegian study data center
REK: Regional committees for medical and health research ethics
SDG: Sustainable development goal
